# GZ17-6.02 and Doxorubicin Interact to Kill Sarcoma Cells via Autophagy and Death Receptor Signaling

**DOI:** 10.3389/fonc.2020.01331

**Published:** 2020-09-02

**Authors:** Laurence Booth, Cameron West, Daniel Von Hoff, Paul Dent

**Affiliations:** ^1^Departments of Biochemistry and Molecular Biology, Virginia Commonwealth University, Richmond, VA, United States; ^2^Genzada Pharmaceuticals, Sterling, KS, United States; ^3^Translational Genomics Research Institute (TGEN), Phoenix, AZ, United States

**Keywords:** autophagy, GZ17-6.02, sarcoma, doxorubicin, CD95, death receptor

## Abstract

GZ17-6.02 (602) is presently under phase I clinical evaluation (NCT03775525). We defined the mechanisms by which it interacted with a standard of care therapeutic doxorubicin to kill sarcoma cells. Doxorubicin and 602 interacted to rapidly activate ATM and c-MET, inactivate mTOR, AKT, and p70 S6K, enhance the expression of Beclin1 and reduce the levels of K-RAS and N-RAS. This was followed later by the drugs interacting to reduce expression of MCL-1, BCL-XL, and HDAC6. Knock down of ATM prevented the drugs alone or in combination inactivating mTOR or activating ULK1. Knock down of c-MET significantly enhanced [doxorubicin + 602] lethality. Knock down of ATM and to a greater extent ULK1, Beclin1, or ATG5 significantly reduced killing by 602 alone or when combined with doxorubicin. Expression of an activated mTOR mutant suppressed killing, autophagosome formation and prevented autophagic flux. In the absence of Beclin1, knock down of CD95, or FADD, or over-expression of c-FLIP-s or BCL-XL abolished tumor cell killing. We conclude that 602 and doxorubicin interact to increase autophagosome formation and autophagic flux as well as causing elevated death receptor signaling resulting in mitochondrial dysfunction and tumor cell death.

## Introduction

GZ17-6.02 (602) comprises of three natural products, curcumin, isovanillin, and harmine (1, 2). In our most recent studies in eight gastrointestinal tumor cell lines we determined that low concentrations of 602 killed tumor cells and interacted with a standard of care drug for these tumor types, 5-fluorouracil (5FU), to further enhance tumor cell death (2). GZ17-6.02 activated an ATM-AMPK signaling module which was responsible for inactivation of mTORC1 and mTORC2, dephosphorylation of ULK1 S757, and increased phosphorylation of ULK1 S317 and of ATG13 S318. Autophagosome formation and autophagic flux were observed. GZ17-6.02 and 5FU caused greater ATM activation, more autophagosome formation and more tumor cell killing; knock down of ULK1, Beclin1, or ATG5 suppressed the lethality of 602 alone and when in combination with 5FU. Exposure of CT26 mouse colorectal tumors to 602 and 5FU prolonged animal survival.

Sarcomas are a diverse set of malignancies originating from mesenchymal cells and these malignancies are approximately one percent of diagnosed human tumors (3, 4). There are two major groups of sarcomas, bone sarcomas and soft tissue sarcomas (STS). Approximately twelve thousand Americans will be diagnosed with a sarcoma in 2020 with ~60% of patients receiving curative surgery and chemo-radiotherapy (5). For the thirty to forty percent of patients whose disease has spread, the majority will ultimately die, despite utilization of the most up-to-date therapeutic modalities.

In the past 5 years the multi-kinase and chaperone inhibitor pazopanib was FDA approved for treatment of the majority of STS subtypes (6). Pazopanib, alone or in combination with other agents in our hands utilizes endoplasmic reticulum stress signaling and autophagosome formation as key components of its mechanisms of action (7–10). In preliminary studies for this manuscript initially we examined whether pazopanib and 602 could interact to kill sarcoma cells. To our surprise, pazopanib and 602 did not interact, with the drug combination causing only approximately a twenty percent increase in killing over either agent individually. Prior to the approval of pazopanib, STS was treated with a variety of well-established cytotoxic chemotherapy regimens, including doxorubicin (11, 12). In further preliminary studies 602 and doxorubicin interacted in an additive to greater than additive fashion to kill STS cells. The present manuscript set out to define the molecular mechanisms by which 602 and doxorubicin interact to kill STS cells.

## Materials and Methods

### Materials

Doxorubicin was purchased from Selleckchem (Houston, TX). GZ17-6.02 was supplied by Genzada Pharmaceuticals Inc. (Sterling, KS). The human sarcoma cell lines HT1080 and MES-SA were obtained from the ATCC (Bethesda, MD) and were not further validated beyond that provided by the vendor. Trypsin-EDTA, DMEM, RPMI, penicillin-streptomycin were purchased from GIBCOBRL (GIBCOBRL Life Technologies, Grand Island, NY). Plasmids were purchased from Addgene, (Cambridge, MA). Commercially available validated short hairpin RNA molecules to knock down RNA/protein levels were from Qiagen (Valencia, CA). The pre-validated smart-pool siRNA molecules used were: siSCR (SI03650318), ATM (SI00604737), cathepsin B (1027416), BAX (GS581), BAK (GS578); AMPKα (GS5562), BIM (GS10018), BAD (GS572), Beclin1 (GS8678), ATG5 (GS9474), CD95 (GS355), AIF (GS9131), eIF2α (GS83939), FADD (GS8772), ULK-1 (GS8408), ATG13 (GS9776). The antibodies used in these studies were: AIF (5318), BAX (5023), BAK (12105), BAD (9239), BIM (2933), BAK1 (12105), Beclin1 (3495), cathepsin B (31718), CD95 (8023), FADD (2782), eIF2α (5324), P-eIF2α S51 (3398), ULK-1 (8054), P-ULK-1 S757 (14202), P-AMPKα T172 (2535), AMPKα (2532), P-ATM S1981 (13050), ATM (2873), ATG5 (12994), mTOR (2983), P-mTOR S2448 (5536), P-mTOR S2481 (2974), ATG13 (13468), MCL-1 (94296), BCL-XL (2764), P-AKT T308 (13038), P-ERK1/2 (5726), P-STAT3 Y705 (9145), P-p65 NFκB S536 (3033), p62 (23214), LAMP2 (49067) all from Cell Signaling Technology; P-ULK-1 S317 (3803a) from Abgent; P-ATG13 S318 (19127) from Novus Biologicals. Antibodies directed against RAS proteins: Thermo-Fisher (Waltham MA) N-RAS PA5-14833; K-RAS PA5-44339. Control studies are presented showing on-target specificity of antibodies to detect total protein levels and phosphorylated levels of proteins (Supplementary Figures 1, 2).

### Methods

#### Culture, Transfection, and *in vitro* Exposure of Cells to Drugs

All cell lines were cultured at 37°C (5% (v/v CO_2_) *in vitro* using RPMI supplemented with 5% (v/v) fetal calf serum and 10% (v/v) non-essential amino acids. Cells were transfected with siRNA molecules or plasmids as described in prior manuscripts (7–10). Cells were transfected with plasmids (0.1 μg) using lipofectamine 2000. Representative data sets showing protein knock down or over-expression are presented in Supplementary Figures 1, 2.

#### Detection of Cell Death by Trypan Blue

Trypan blue exclusion was used to assess cell viability at each experimental time point. Floating cells were isolated along with attached cells that were harvested by trypsinization with Trypsin/EDTA for ~3 min at 37°C. Following isolation, the total cell population for each experimental point was assessed for cell viability.

#### Colony Formation Assays

Single cells were plated into 60 mm dishes (500/dish). Twelve hour after plating cells were treated with vehicle control, 602 (0.5–2.0 μM), doxorubicin (0.5–2.0 μM) or the drugs in combination at a fixed dose ratio. Twenty-four hour after drug exposure, the growth media was removed, cells washed with drug free media and then cells were cultured for an additional 7 days in drug free media. Cells were fixed in place, stained with crystal violet and the number of colonies, a group of > 50 cells, counted. The synergy of drug interaction was determined via the Method of Cho and Tallalay using Calcusyn for Windows program.

#### Detection of Protein Expression and Protein Phosphorylation by In-Cell Western Blotting Using a Hermes WiScan Microscope

The Hermes WiScan wide field microscope (https://idea-bio.com/products/wiscan-hermes/). The machine combines high quality optics with a high-quality computer driven microscope stage, and with dedicated software, for example, to analyze the immunofluorescent staining intensity of individual cells, i.e., *true* in-cell western blotting. Cells (4 × 10^3^) were plated into 96-well plates and allowed to grow over night. A typical experiment proceeds thus: three independent thaws/cultures of a particular tumor cell type are sub-cultured into individual 96-well plates. Twenty-four hour after plating, the cells are transfected with a control plasmid or a control siRNA, or with plasmids to express various proteins or validated siRNA molecules to knock down the expression of various proteins. After another 24 h, the cells are ready for drug exposure(s). At various time-points after the initiation of drug exposure, cells are fixed in place with permeabilization. Standard immunofluorescent blocking procedures are employed, followed by incubation of different wells with a variety of validated primary antibodies. The next morning, after washing, fluorescent-tagged secondary antibodies are added to each well; in general, we have found that using more than two tagged antibodies in each well results in poorer data / image quality. After 3 h of incubation, the secondary antibody is removed, the cells washed again, and are hydrated with phosphate buffered saline prior to microscopic examination. Based on the experiment, cells are visualized at either 10X magnification for bulk assessments of immunofluorescent staining intensity or at 60X magnification for assessments of protein or protein-protein colocalization.

For studies at 10X magnification, the operator selects which fluorescent antibody will be assessed first, i.e., in the red or green channel, and then focuses the microscope in a vehicle control transfection control well. The operator then outlines for the computer controlling the microscope “what is a cell.” In other words, the operator manually inputs the criteria for each specific tumor cell line segregating away detection of what is obvious debris or a staining artifact. The operator then sets how many cells per well are to be assessed for their immunofluorescent staining intensity; 100. The computer/microscope then determines the background fluorescence in the well and in parallel randomly determines the mean fluorescent intensity of those 100 cells; the operator is provided with this mean intensity value. Of note for scientific rigor is that the operator does not personally manipulate the microscope to examine specific cells; the entire fluorescent accrual method is independent of the operator. Once the entire plate has been scanned for one of the secondary antibodies, the second secondary antibody with a different fluorescence range can similarly be used to define the mean intensity value in each well.

Once data from the first independent set of plated cells has been obtained, the second and third sets of independent plated cells can be processed through the machine. Thus, we obtain three independent sets of fluorescence data from the three individual cultures, with an independent total of 300 cells under each condition being assessed (+/– SD).

### Data Analysis

Comparison of the effects of various treatments (in independent triplicate three times) was using one-way ANOVA and a two tailed Student's *t*-test. Differences with a *p* < 0.05 were considered statistically significant. Experiments are the means of multiple individual points from multiple experiments (± SD).

## Results

### GZ17-6.02 and Doxorubicin Interact to Kill Sarcoma Cells

**Figure 1:**
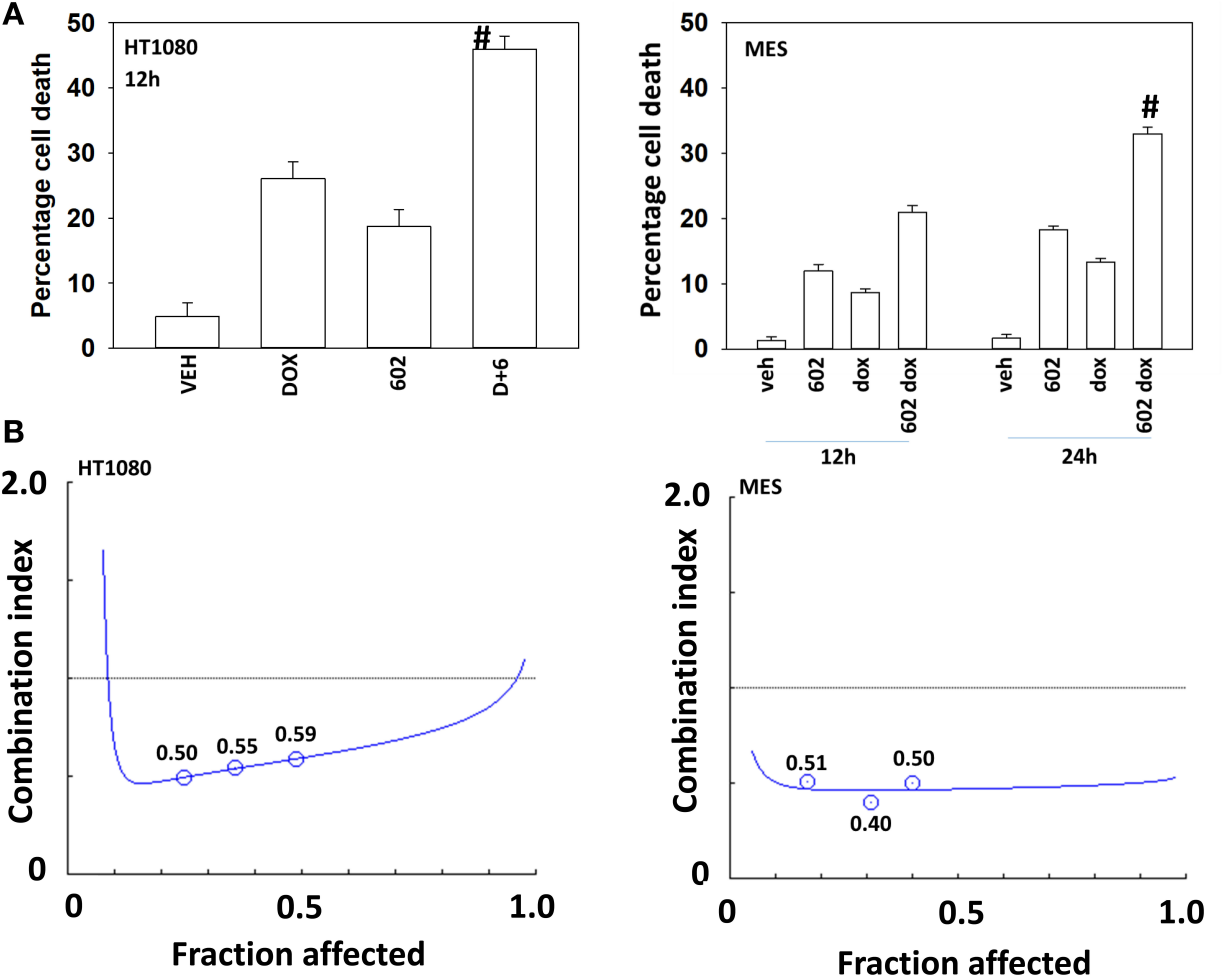
GZ17-6.02 and doxorubicin interact to kill sarcoma cells. **(A)** HT1080 and MES human sarcoma cells were treated with vehicle control, GZ17-6.02 (2 μM final curcumin), doxorubicin (200 nM) or the drugs in combination for 12–24 h. Cells were isolated, and viability determined by a trypan blue exclusion assay (*n* = 3 +/–SD). ^#^*p* < 0.05 greater than either 602 or doxorubicin alone. **(B)** Single cells were plated into 60 mm dishes (500/dish, six separate dishes from two independent thawed cell vials). Twelve hour after plating cells were treated with vehicle control, 602 (0.5–2.0 μM), doxorubicin (0.5–2.0 μM) or the drugs in combination at a fixed dose ratio. Twenty-four hour after drug exposure, the growth media was removed, cells washed with drug free media and then cells were cultured for an additional 7 days in drug free media. Cells were fixed in place, stained with crystal violet and the number of colonies, a group of >50 cells, counted. The synergy of drug interaction was determined via the Method of Cho and Tallalay using Calcusyn for Windows program. A combination index (CI) value of <1.0 is considered synergistic.

Our preliminary studies demonstrated that GZ17-6.02 (602) interacted with doxorubicin to kill HT1080 cells (Figure 1A). Exposure of MES cells to 602 alone caused a similar amount of tumor cell death when compared to prior studies in GI tumor cells, whereas HT1080 cells appeared to be more sensitive (2). In colony formation assays 602 and doxorubicin synergized to kill sarcoma cells with combination index values below ~1.0 (Figure 1B).

### GZ17-6.02 and Doxorubicin Interact to Activate ATM, Inactivate AKT and mTOR, and Reduce RAS Protein Levels, Concomitant With Activating Cyto-Protective c-MET

**Figure 2:**
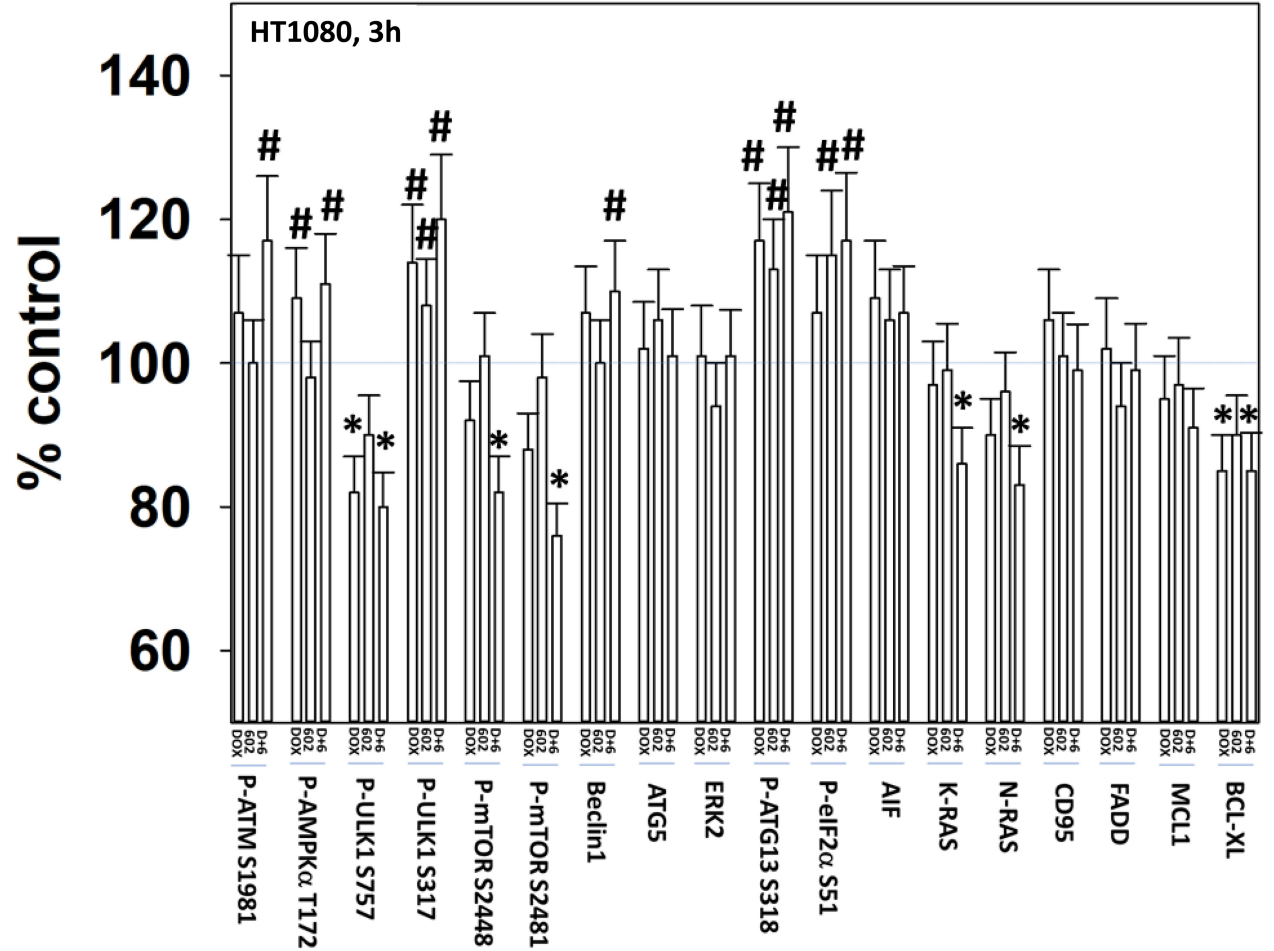
GZ17-6.02 and doxorubicin interact after a 3 h exposure to activate an ATM-AMPK-ULK1- autophagy pathway concomitant with inactivation of mTOR and increased Beclin1 expression. HT1080 human sarcoma cells were treated with vehicle control, GZ17-6.02 (2 μM final curcumin), doxorubicin (200 nM) or the drugs in combination for 3 and 6 h. Cells were fixed in place and immunostained to detect the total expression and the total phosphorylation of the indicated proteins. The phosphorylation of each phosphoprotein was corrected for total protein expression; non-phosphoproteins had their expression corrected using invariant ERK2 expression. (*n* = 3 +/–SD) *p < 0.05 less than vehicle control; ^#^*p* < 0.05 greater than vehicle control.

**Figure 3:**
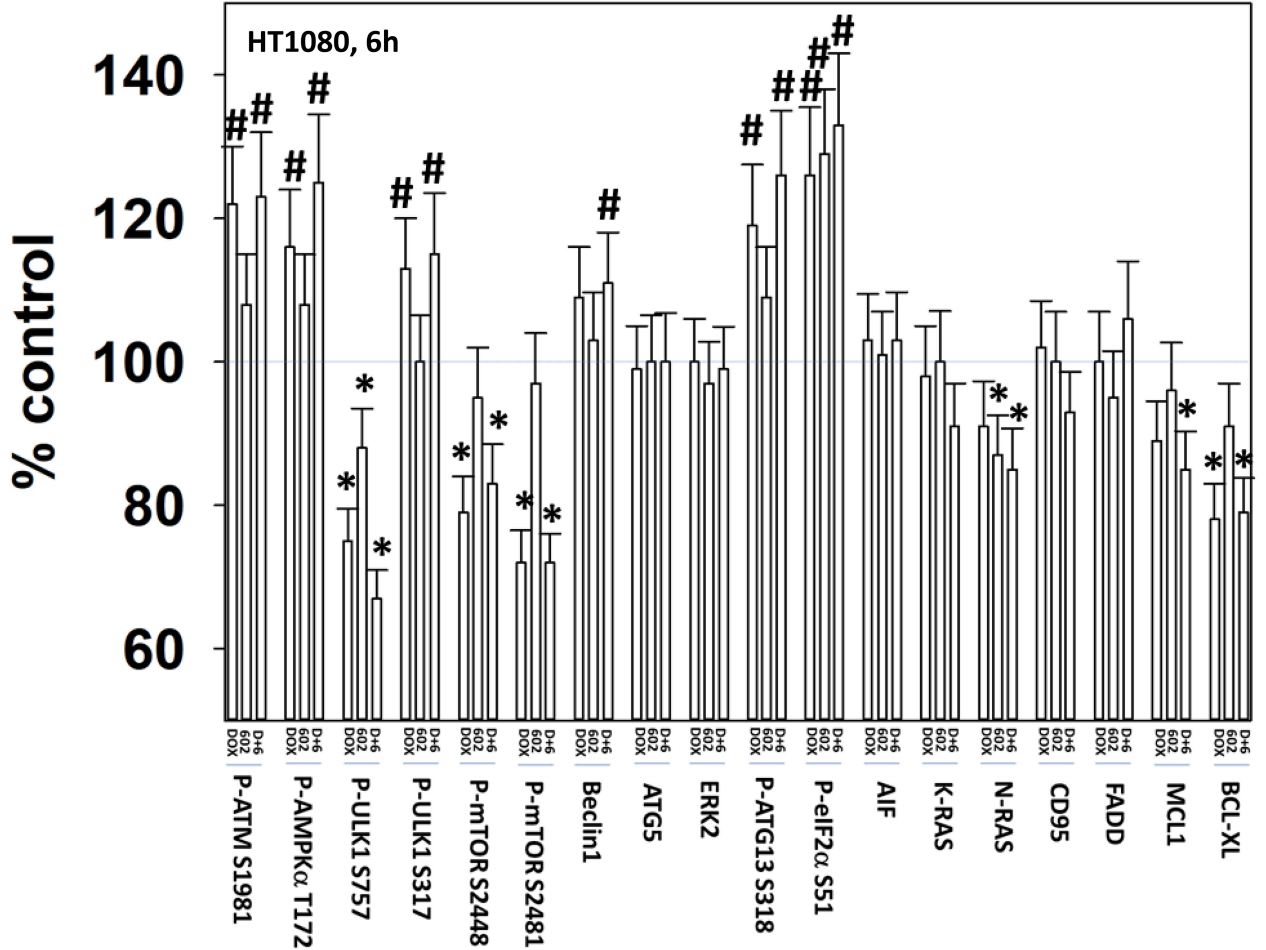
GZ17-6.02 and doxorubicin interact after a 6 h exposure to activate an ATM-AMPK-ULK1- autophagy pathway concomitant with inactivation of mTOR and increased Beclin1 expression. HT1080 human sarcoma cells were treated with vehicle control, GZ17-6.02 (2 μM final curcumin), doxorubicin (200 nM) or the drugs in combination for 3 and 6 h. Cells were fixed in place and immunostained to detect the total expression and the total phosphorylation of the indicated proteins. The phosphorylation of each phosphoprotein was corrected for total protein expression; non-phosphoproteins had their expression corrected using invariant ERK2 expression. (*n* = 3 +/–SD) *p < 0.05 less than vehicle control; ^#^*p* < 0.05 greater than vehicle control.

**Figure 4:**
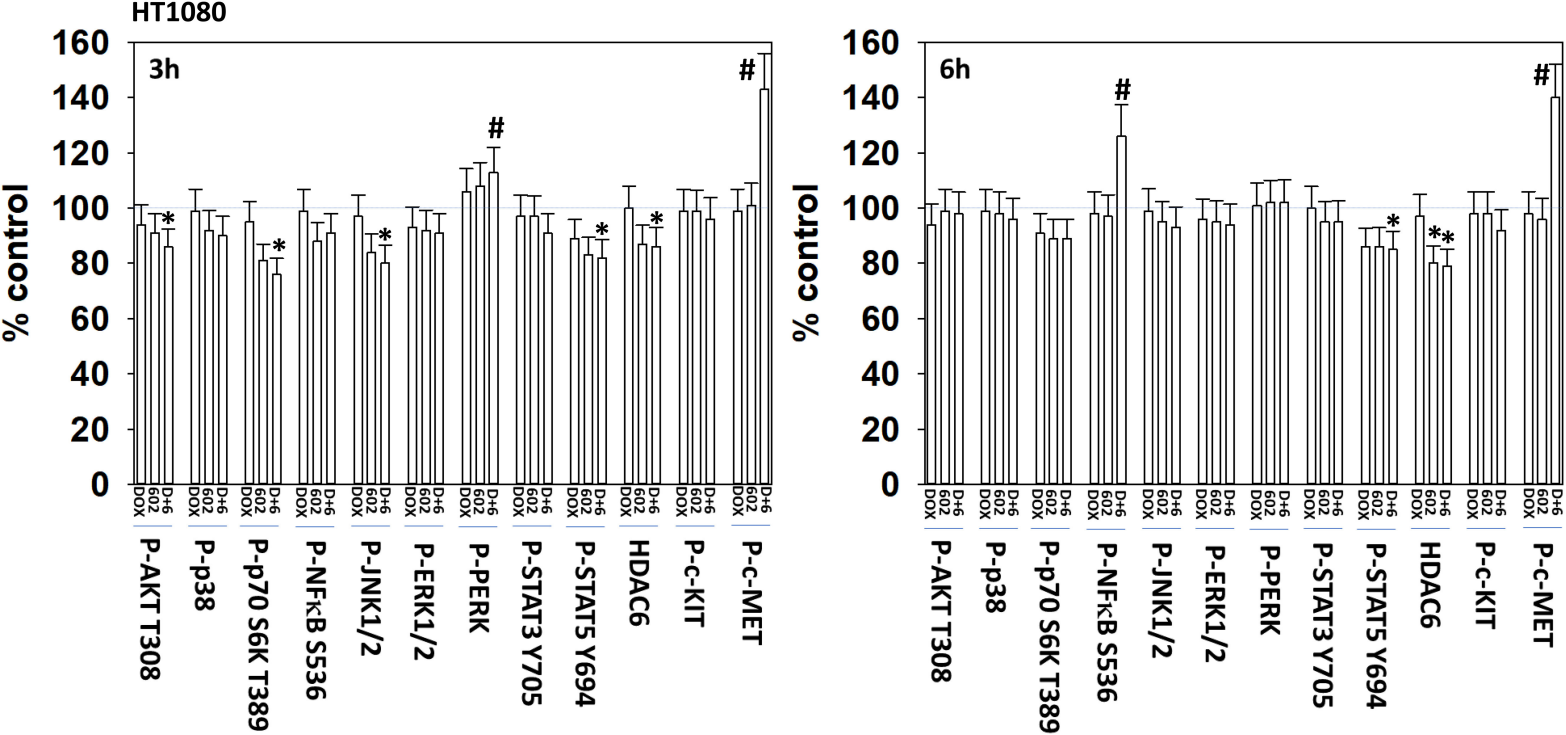
GZ17-6.02 and doxorubicin interact to activate c-MET. HT1080 human sarcoma cells were treated with vehicle control, GZ17-6.02 (2 μM final curcumin), doxorubicin (200 nM) or the drugs in combination for 3 and 6 h. Cells were fixed in place and immunostained to detect the total expression and the total phosphorylation of the indicated proteins. The phosphorylation of each phosphoprotein was corrected for total protein expression; HDAC6 had its expression corrected using invariant ERK2 expression. (*n* = 3 +/–SD) *p < 0.05 less than vehicle control; ^#^*p* < 0.05 greater than vehicle control.

We next defined the impact of 602 in the presence or absence of doxorubicin on cellular signaling processes. As a single agent over a 6-h time course, 602 activated ataxia telangiectasia mutated (ATM), the AMP-dependent protein kinase (AMPK), ULK1, inhibited mTORC1, mTORC2 and eIF2α, and increased ATG13 S318 phosphorylation (Figures 2, 3; Supplementary Figure 3). Most obviously, at the 3-h timepoint, 602 and doxorubicin interacted in a greater than additive fashion to: activate ATM; inactivate AKT, p70 S6K, JNK1/2, mTORC1, mTORC2, and STAT5; increase Beclin1 expression and reduce the levels of HDAC6, K-RAS, and N-RAS; and activate the receptor tyrosine kinase c-MET (Figure 4; Supplementary Figure 4). Knock down of the hepatocyte growth factor receptor, c-MET, significantly enhanced the lethality of [602 + doxorubicin] in sarcoma cells, arguing that the immediate-early activation of this receptor tyrosine kinase was a compensatory survival signal (Supplementary Figure 5). There are at present multiple small molecule agents/drugs that have been developed to inhibit c-MET, including crizotinib (XALKORI®) that is FDA approved for the treatment of ALK or ROS1 mutant expressing non-small cell lung cancer. We determined whether any c-MET inhibitor could enhance [602 + doxorubicin] lethality (Supplementary Figure 6). The standard of care sarcoma drug pazopanib was toxic as a single agent and enhanced the lethality of [602 + doxorubicin]. The c-MET inhibitors crizotinib, foretinib, BMS-777607 and tivantinib all also enhanced basal levels of tumor cell death and enhanced [602 + doxorubicin] lethality.

### Cyto-Protective Activation of c-MET Requires Its Ligand HGF

In prior radiation oncology-based studies we have demonstrated that ionizing radiation, in carcinoma cells, causes two waves of ERBB1 and ERK1/2 activation, the second wave being caused by autocrine TGFα signaling (13–15). As we observed [602 + doxorubicin] causing activation of c-MET, we hypothesized that the ligand for this receptor, hepatocyte growth factor (HGF), may be playing a role in the receptor's compensatory survival signal. Cells were exposed to [602 + doxorubicin] in the presence of a control antibody or an antibody to neutralize HGF. Antibody-based neutralization of HGF function prevented the drug-induced activation of c-MET (Supplementary Figures 7, 8).

### GZ17-6.02 and Doxorubicin Interact to Kill via Activation of ATM-AMPK-ULK1-Autophagy

**Figure 5:**
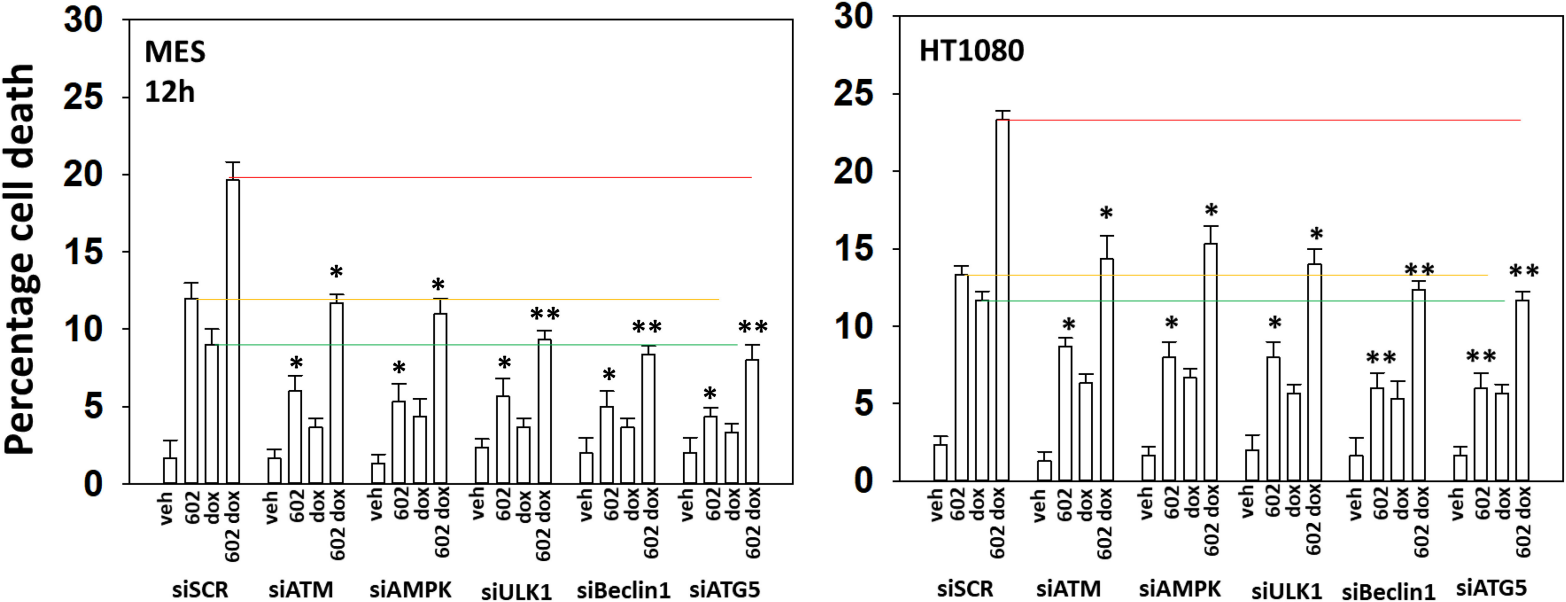
GZ17-6.02 and doxorubicin individual and combination lethality is suppressed by knock down of ULK1, Beclin1, or ATG5. HT1080 and MES human sarcoma cells were transfected with a scrambled siRNA control (siSCR) or siRNA molecules to knock down the indicated proteins. Twenty-four hour afterwards, cells treated with vehicle control, GZ17-6.02 (2 μM final curcumin), doxorubicin (200 nM) or the drugs in combination for 12 h. Cells were isolated, and viability determined by a trypan blue exclusion assay (*n* = 3 +/–SD). *p < 0.05 less than vehicle control; **p < 0.05 less than corresponding value in siATM or siAMPKα transfected cells. The red, yellow and green lines are included for purposes of comparison between the individual treatments and each of the specific transfections.

Our cell signaling data with 602 and doxorubicin in sarcoma cells was similar, though not identical, to our prior work with 602 in GI tumor cells (2). Knock down of ATM prevented the drug combination from activating the AMPK and ULK1, inactivating mTORC1 or increasing ATG13 S318 phosphorylation (Supplementary Figure 9). Knock down of either ATM or AMPKα significantly reduced the lethality of 602 as a single agent and when it was combined with doxorubicin (Figure 5). Knock down of Beclin1 or ATG5, and in one cell line ULK1, suppressed killing to a greater extent than observed in ATM/AMPKα knock down cells. This data collectively tends to emphasize the importance of “autophagy” in the lethality of [602 + doxorubicin].

### GZ17-6.02 and Doxorubicin Interact to Cause Toxic Autophagic Flux

**Figure 6:**
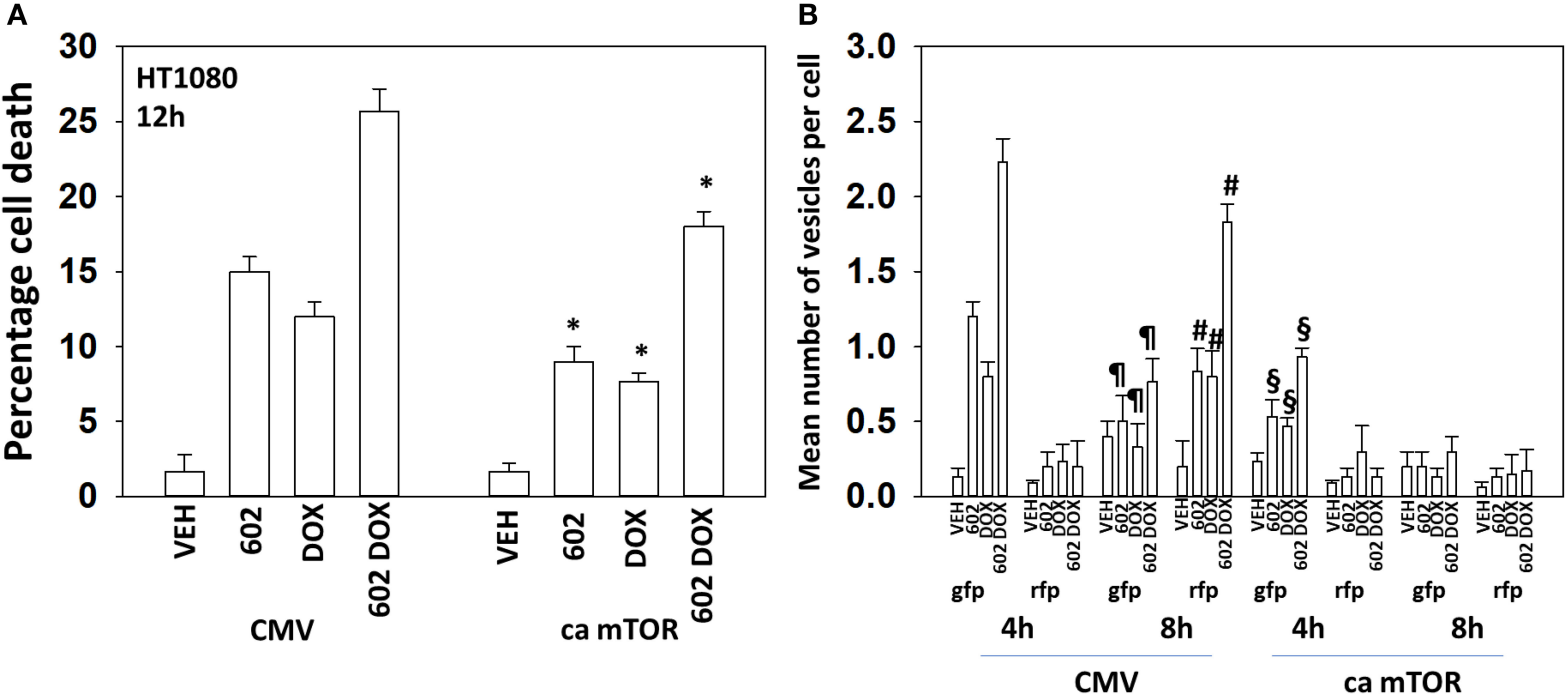
Expression of activated mTOR suppresses tumor cell killing and abolishes autophagic flux. **(A)** HT1080 human sarcoma cells were transfected with an empty vector plasmid (CMV) or with a plasmid to express a mutant active mTOR protein. Twenty-four hour afterwards, cells treated with vehicle control, GZ17-6.02 (2 μM final curcumin), doxorubicin (200 nM) or the drugs in combination for 12 h. Cells were isolated, and viability determined by a trypan blue exclusion assay (*n* = 3 +/–SD). *p < 0.05 less than vehicle control. **(B)** HT1080 cells were transfected with an empty vector plasmid (CMV) or with a plasmid to express a mutant active mTOR protein and in parallel all transfected with a plasmid to express LC3-GFP-RFP. Twenty-four hour afterwards, cells treated with vehicle control, GZ17-6.02 (2 μM final curcumin), doxorubicin (200 nM) or the drugs in combination for 4 or 8 h. At each time point the mean number of intense GFP+ and RFP+ vesicles was determined counting > 40 cells per condition. (*n* = 3 +/–SD) ^#^*p* < 0.05 greater than corresponding values after 4 h; ^¶^*p* < 0.05 less than corresponding values after 4 h; ^§^*p* < 0.05 less than corresponding values in CMV transfected cells.

The kinase mTOR is a key gate-keeper regulator and signal integrator controlling the formation of autophagosomes. Expression of an activated mutant form of mTOR significantly reduced the lethality of 602, doxorubicin and the drug combination, though the effect was less dramatic than observed for knocking down Beclin1 or ATG5 (Figure 6A; Supplementary Figure 10). We then determined the impact of activated mTOR on the ability of cells to form autophagosomes and exhibit autophagic flux. Doxorubicin and 602 combined in an at least additive fashion to cause GFP+ autophagosome formation within 4 h with no alteration in RFP+ autolysosome formation (Figure 6B; Supplementary Figure 10). Eight hours after drug exposure, the numbers of GFP+ autophagosome levels declined and those of RFP+ autolysosome formation had increased. This implies autophagic flux had occurred. In cells expressing activated mTOR, the formation of autophagosomes after 4 h was reduced compare to empty vector transfected cells. And, after 8 h, no statistically significant increase in autolysosome levels was observed, despite autophagosome levels declining. Collectively, our data argue that the expression of autophagy regulatory proteins, Beclin1 and ATG5, together with inactivation of mTOR, play key roles in mediating at least fifty percent of the anti-cancer actions of [602 + doxorubicin].

### GZ17-6.02 and Doxorubicin Interact to Cause Toxic Death Receptor Signaling

**Figure 7:**
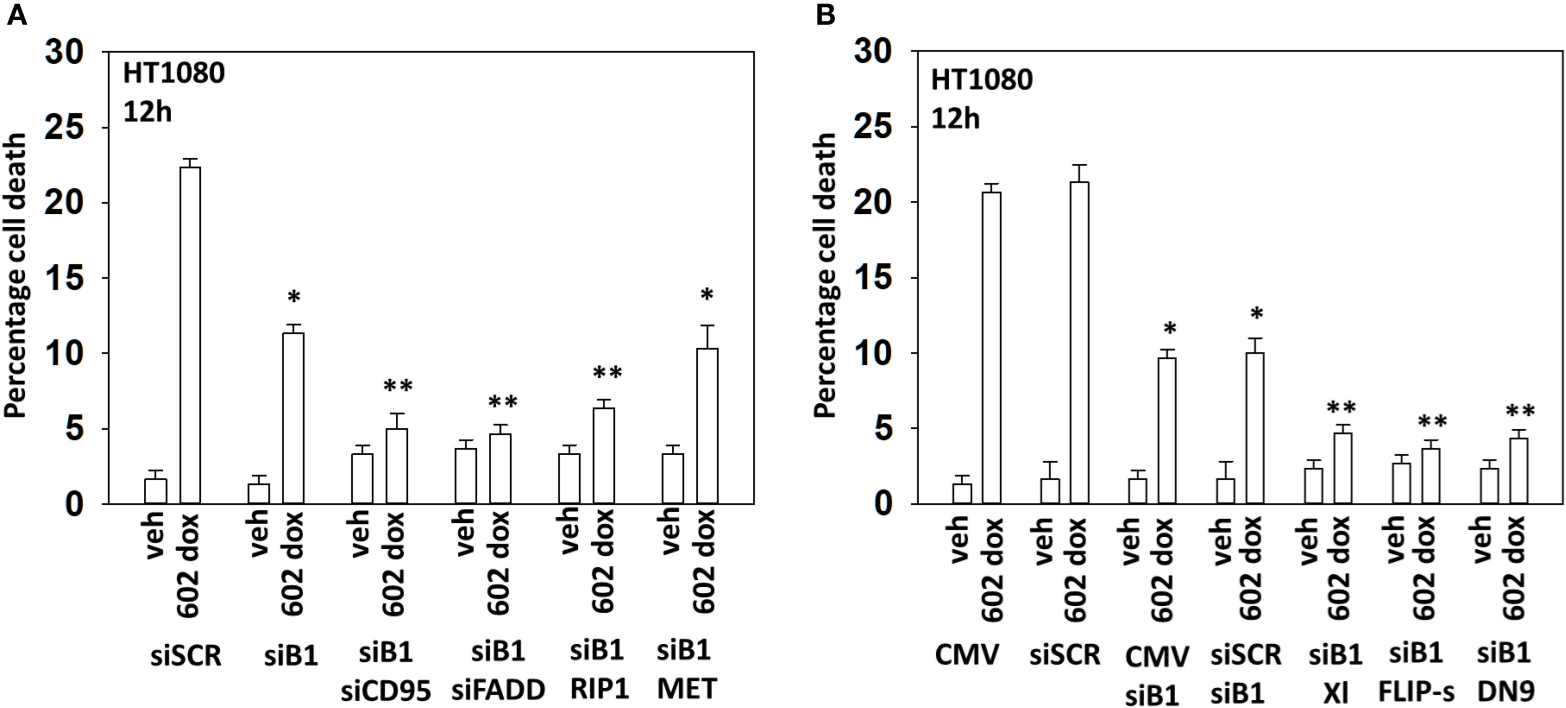
In the absence of autophagy, GZ17-6.02 -induced killing is mediated via CD95-FADD. **(A)** HT1080 human sarcoma cells were transfected with a scrambled siRNA (siSCR) or with an siRNA to knock down the expression of Beclin1, and in parallel, siBeclin1 transfected cells were transfected to knock down the expression of the indicated proteins. Twenty-four hour afterwards, cells treated with vehicle control or with [GZ17-6.02 (2 μM final curcumin) + doxorubicin (200 nM)] in combination for 12 h. Cells were isolated, and viability determined by a trypan blue exclusion assay (*n* = 3 +/–SD). *p < 0.05 less than vehicle control; **p < 0.05 less than corresponding value in siBeclin1 alone. **(B)** HT1080 cells were transfected with a scrambled siRNA (siSCR) or with an siRNA to knock down the expression of Beclin1, and in parallel, cells were transfected with an empty vector plasmid or with plasmids to express BCL-XL, c-FLIP-s or dominant negative caspase 9. Twenty-four hour afterwards, cells treated with vehicle control or with [GZ17-6.02 (2 μM final curcumin) + doxorubicin (200 nM)] in combination for 12 h. Cells were isolated, and viability determined by a trypan blue exclusion assay (*n* = 3 +/–SD). *p < 0.05 less than vehicle control; **p < 0.05 less than corresponding value in siBeclin1 alone.

We next wished to determine the additional autophagy-independent mechanisms that [602 + doxorubicin] recruit to kill sarcoma cells. In cells with the expression of Beclin1 transiently reduced, knock down of CD95, FADD, or RIP1 almost abolished tumor cell killing (Figure 7A; Supplementary Figure 11). Previously, we had shown that knock down of c-MET enhanced [602 + doxorubicin] lethality; however, knock down of c-MET in the absence of Beclin1 did not increase killing arguing that activation of c-MET protects cells by trying to restrain autophagy. Over-expression of the caspase 8 inhibitor c-FLIP-s, the mitochondrial protective protein BCL-XL or a dominant negative form of caspase 9 also virtually abolished drug toxicity when Beclin1 expression was knocked down (Figure 7B; Supplementary Figure 11). Thus, there are two major components of cell killing, one requiring autophagy and the other death receptor signaling. Data in Figure 5 shows us that knock down of Beclin1 or ATG5 is strongly protective. Our published data has shown that 602 uses death receptor signaling as a key component of the killing process and data in Figure 7 shows us that the majority of the killing caused by the [602 + doxorubicin] combination is prevented by knock down of Beclin1 combined with interdiction of CD95 death receptor signaling. Hence, the two most important mechanisms by which 602 and doxorubicin combine to kill sarcoma cells are via autophagosome formation and activation of death receptors.

### GZ17-6.02 Reduced the Expression of HDAC Proteins and Alters Their Sub-cellular Localization

**Figure 8:**
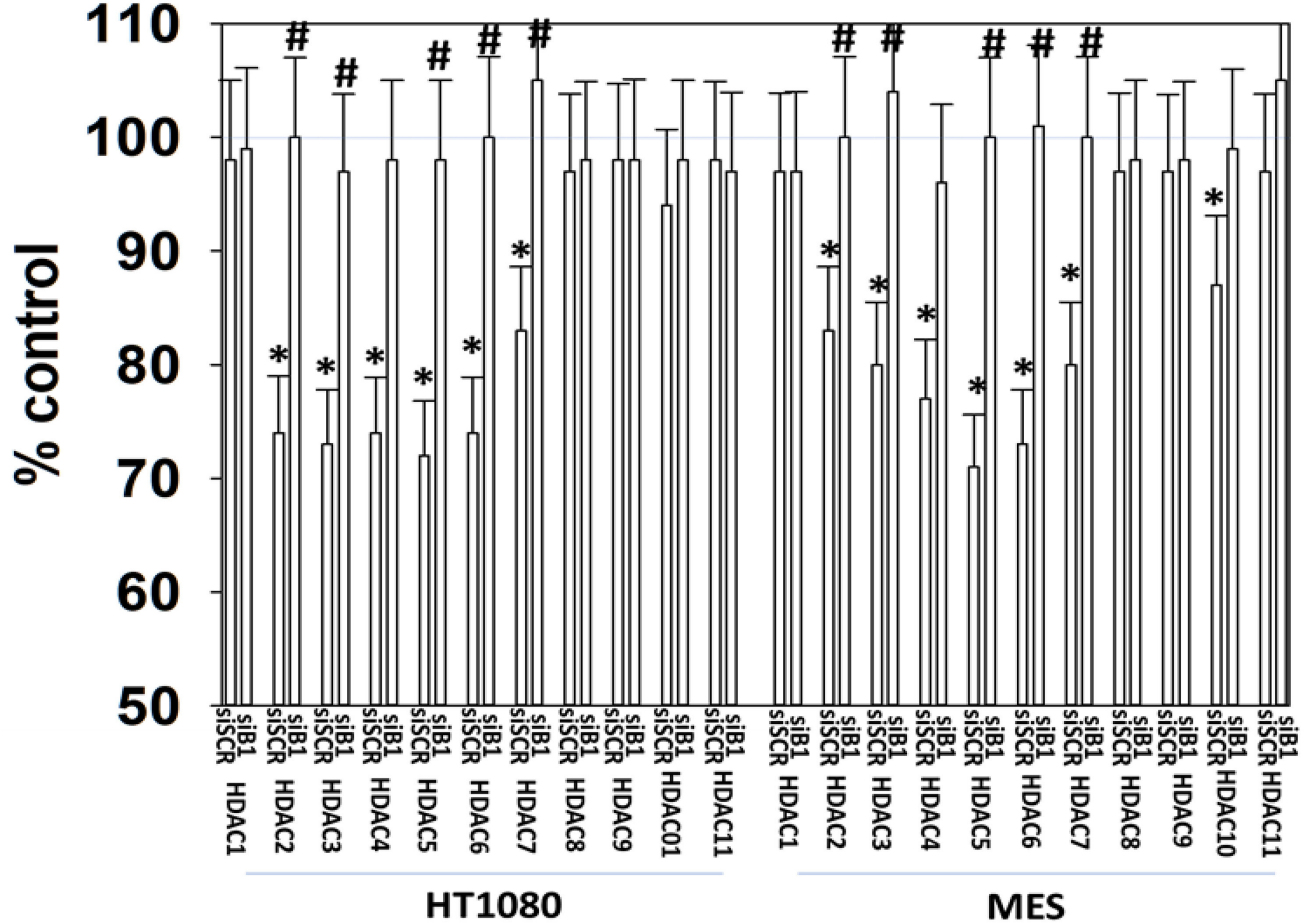
GZ17-6.02 regulates the expression and location of HDAC proteins. HT1080 and MES human sarcoma cells were transfected with a scrambled siRNA (siSCR) or with an siRNA to knock down the expression of Beclin1. Twenty-four hour afterwards, cells treated with vehicle control or with [GZ17-6.02 (2 μM final curcumin) for 6 h. Cells were fixed in place and immunostaining performed to detect the expression of HDACs1–11 and total ERK2 (*n* = 3 +/–SD). *p < 0.05 less than vehicle control; ^#^*p* < 0.05 greater than vehicle control.

**Figure 9:**
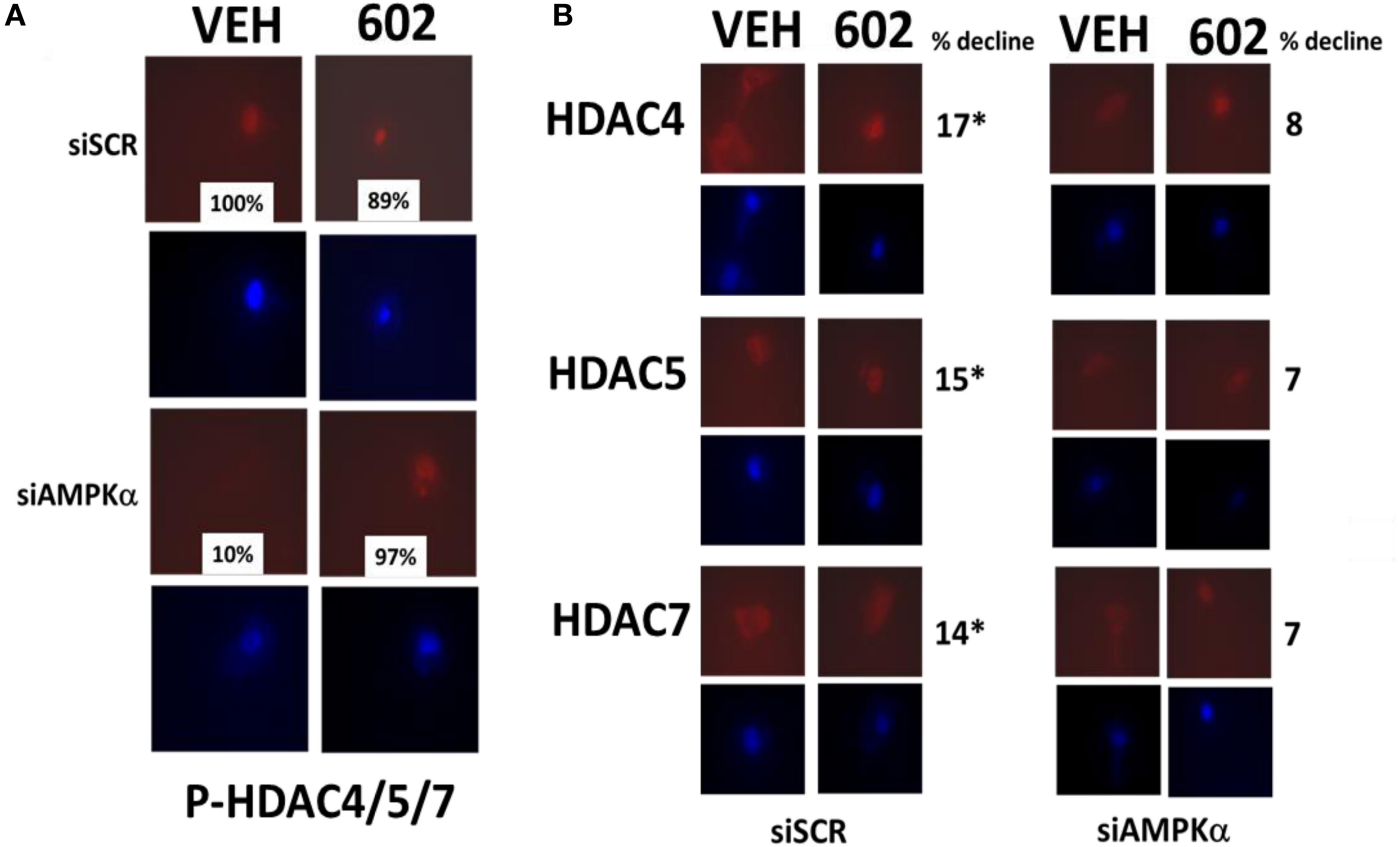
GZ17-6.02 regulates the expression and location of HDACs. **(A,B)** HT1080 cells were transfected with a scrambled siRNA (siSCR) or with an siRNA to knock down the expression of AMPKα. Twenty-four hour afterwards, cells treated with vehicle control or with [GZ17-6.02 (2 μM final curcumin) 1 h. Cells were fixed in place and immunostaining performed to detect the expression of HDACs4/5/7 and P-HDACs4/5/7 and total ERK2 (*n* = 3 +/–SD). *p < 0.05 less than vehicle control (*n* = 3 +/–SD). *p < 0.05 less than vehicle control.

**Figure 10:**
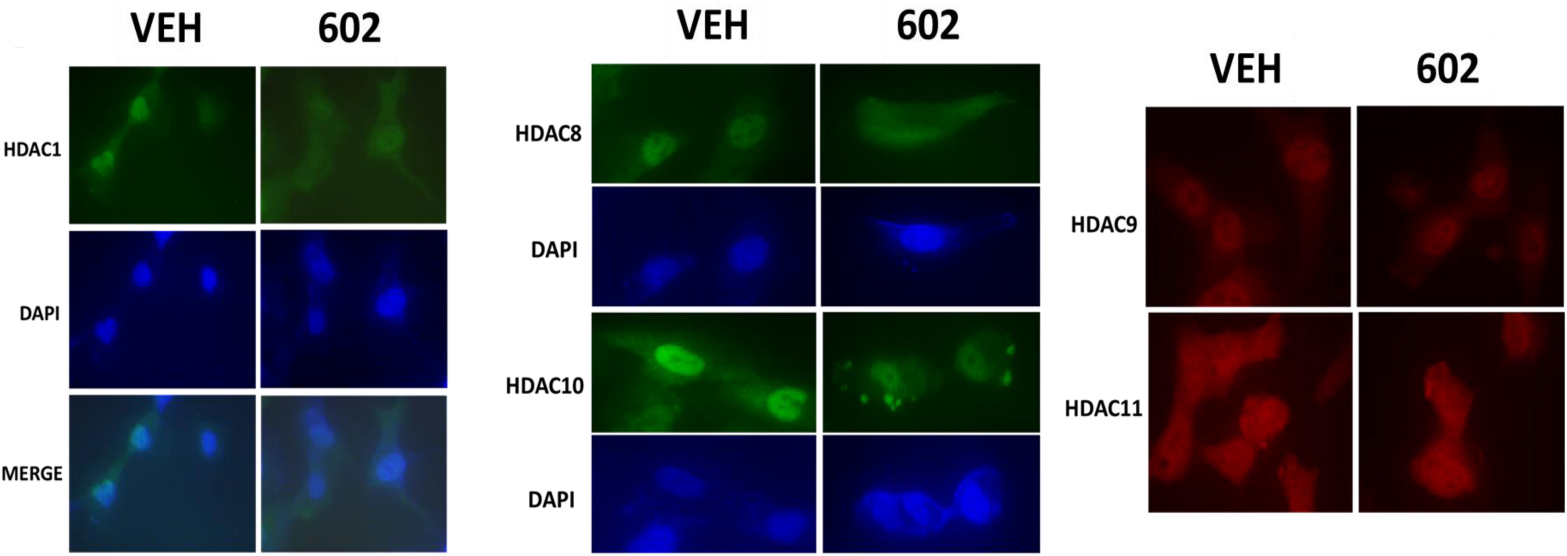
GZ17-6.02 causes some HDACs to leave the nucleus; some to enter the nucleus and some do not move. HT1080 cells were transfected with a scrambled siRNA (siSCR) or with an siRNA to knock down the expression of AMPKα. Twenty-four hour afterwards, cells treated with vehicle control or with [GZ17-6.02 (2 μM final curcumin) 1 h. Cells were fixed in place and immunostaining performed to detect the expression and localization of HDACs1/8/9/10/11.

**Figure 11:**
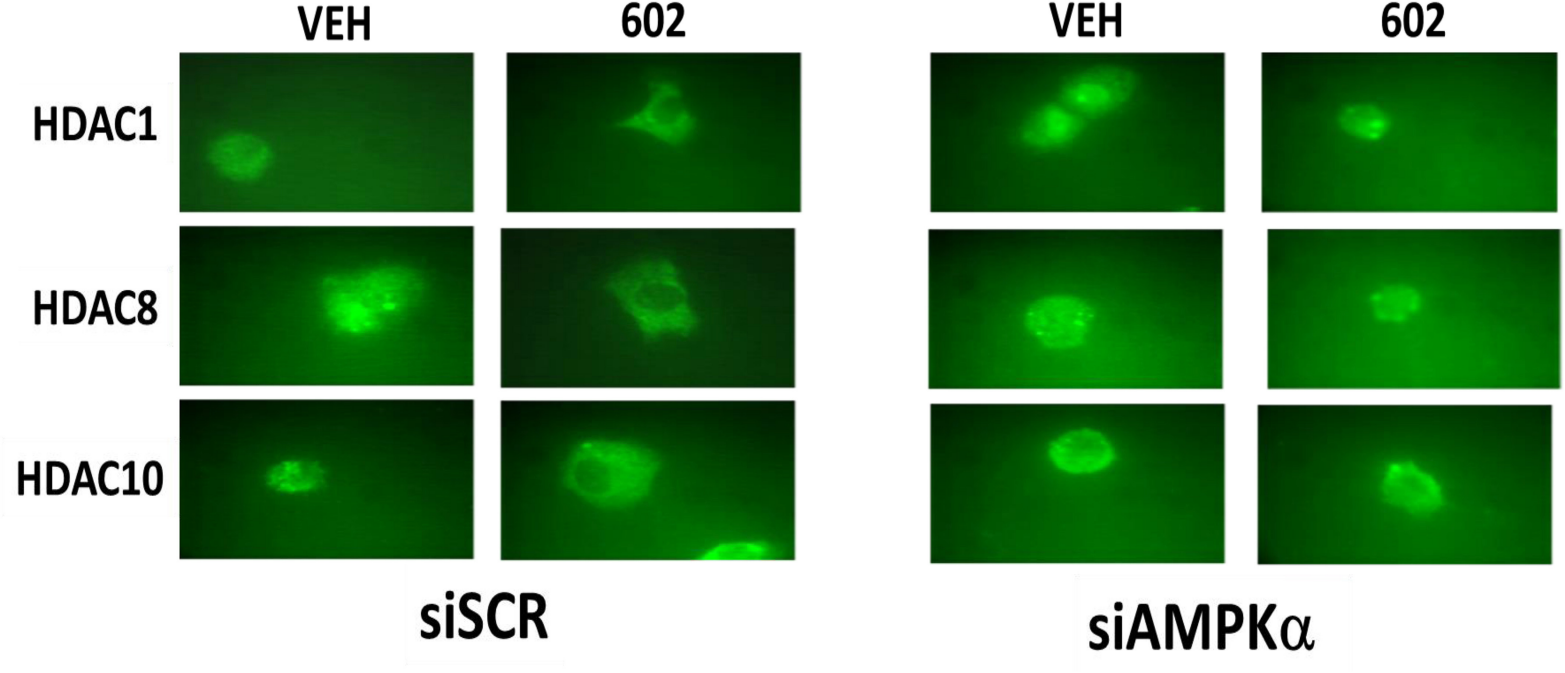
Nuclear exit of HDACs1/8/10 requires signaling by the AMPK. HT1080 cells were transfected with a scrambled siRNA (siSCR) or with an siRNA to knock down the expression of AMPKα. Twenty-four hour afterwards, cells treated with vehicle control or with [GZ17-6.02 (2 μM final curcumin)] 1 h. Cells were fixed in place and immunostaining performed to detect the expression and localization of HDACs1/8/10.

It has been argued that 602 directly alters tumor cell biology via the regulation of super-enhancer elements (16). Exposure of sarcoma cells to 602 for 1 h reduced the expression of histone deacetylases (HDACs) 1–11, an effect which was prevented by knock down of Beclin1 (Figure 8). HDAC proteins can be phosphorylated by the AMPK which alters their sub-cellular localization; phosphorylated HDACs leave the nucleus (17–19). In sarcoma cells, a 1 h 602 exposure caused increased phosphorylation of the conserved phosphorylation site in HDACs4/5/7, an effect which required expression of AMPKα (Figure 9A). This was associated with the significant majority of HDAC4/5/7 *concentrating* in the nucleus (Figure 9B; ^*^p < 0.05). HDACs1/8/9/10/11 under basal conditions were all localized in the nucleus (Figure 10). After treatment of cells with 602, HDACs1/8/10 exited the nucleus, however, the localization of HDACs9/11 was not changed (19). Knock down of AMPKα prevented 602 from translocating HDACs1/8/10 out of the nucleus (Figure 11). Thus, in sarcoma cells, AMPK signaling caused by 602 exposure regulates HDAC degradation, HDAC nuclear entry and HDAC nuclear exit.

## Discussion

The present studies were performed to determine the biology of sarcoma cells when exposed to GZ17-6.02. GZ17-6.02 as a single agent caused tumor cell death to a similar degree as was previously noted in GI tumor cells, and when 602 was combined with a former standard of care agent for the treatment of sarcoma, doxorubicin, additional cell killing was observed (2).

The drugs combined to activate an ATM-AMPK-ULK1-autophagy pathway, also inactivating AKT and mTOR, but surprisingly also activating the receptor tyrosine kinase c-MET. Knock down of c-MET or use of c-MET small molecule inhibitors significantly enhanced [602 + doxorubicin] lethality demonstrating that this rapid activation of c-MET was a compensatory survival signal. Activation of c-MET required autocrine signaling by its ligand HGF. Previously, in sarcoma cells treated with the multi-kinase inhibitor pazopanib and the HDAC inhibitors sodium valproate or entinostat we also observed, in drug-resistant cells, that c-MET as well as ERBB1 had become activated (10). Use of the multi-kinase inhibitor neratinib reduced the protein levels of both receptors and significantly enhanced [pazopanib + entinostat] lethality. In colon cancer cells and tumors previously exposed to curcumin combined with sildenafil we discovered that the cells had evolved to express higher levels of PDGFβ concomitant with elevated PDGFRβ phosphorylation in the tumor cells (20, 21). Inhibition of PDGFRβ signaling via siRNA knock down or using the standard of care drug regorafenib restored the efficacy of the curcumin sildenafil combination.

In the studies with pazopanib, entinostat and neratinib, one essential process for efficient tumor cell killing was autophagosome formation and autophagic flux (10). In our present studies with 602 and doxorubicin, the drugs combined to cause greater expression of Beclin1 and to reduce mTORC1 and mTORC2 activities. This was associated with an additive to greater than additive increase in autophagosome levels and with greater autophagic flux. Expression of activated mTOR abolished autophagic flux and significantly reduced the lethality of either drug alone or in combination. In a delayed fashion, the drugs interacted to reduce expression of MCL-1, BCL-XL, and HDAC6. Reduced HDAC6 expression will result in higher levels of acetylated and inactive HSP90; HSP90 acts as a chaperone for many proteins including ERBB1 and BCL-XL (9). Knock down of ATM prevented the drugs alone or in combination inactivating mTOR or activating ULK1. Knock down of ATM and to a greater extent ULK1, Beclin1, or ATG5 significantly reduced killing by 602 alone or when combined with doxorubicin. This strongly suggests that activation of ATM plays a critical role in mediating the biological actions of 602 in sarcoma. Expression of an activated mTOR mutant suppressed killing, autophagosome formation and prevented autophagic flux. In the absence of Beclin1, knock down of CD95 or FADD, or over-expression of c-FLIP-s or BCL-XL abolished tumor cell killing. Thus, we have defined the two key mechanisms, autophagy and death receptor signaling, by which 602 alone or in combination with doxorubicin kills sarcoma cells.

In prior studies using combinations of “targeted agents” such as neratinib, afatinib, HDAC inhibitors, sorafenib, and pemetrexed, we were able to demonstrate that cells evolved to express activated growth factor receptors including ERBB1, ERBB3, c-KIT, c-MET, and PDGFRβ (22–24). However, in those studies, the altered / evolved signaling parameters were observed days and weeks following drug exposure, not within several hours as in our present work. In the present studies we are using a topoisomerase poison (doxorubicin) and 602 contains the chemical harmine. Harmine is a putative DNA damaging agent and has been proposed to inhibit the relaxation activity of DNA topoisomerase I and II, and inhibit drug efflux pumps (25, 26). In *Saccharomyces cerevisiae* harmine caused crossing-over and frameshift mutations (27). Yeast mutants that were defective in nucleotide excision repair, in error-prone repair and in recombinational repair all exhibited enhanced sensitivity after exposure to harmine, with double mutants of Rad1/Rad6 showing that NER and error-prone repair are independently involved in repair of harmine-induced DNA lesions. Harmine can elevate micronuclei levels in eukaryotic cells and in the Ames test, harmine could induce frameshift mutations in S. typhimurium strains TA97 and TA98 (28). Thus, the cellular response to 602 and doxorubicin will be the sensing and the outcomes of multiple different forms of DNA damage.

GZ17-6.02 has been argued to regulate super-enhancer regions in the genome which drive a high level of transcription of transcriptional regulatory proteins (16). The authors in reference 16 observed reduced Histone 3 K27 acetylation 24–72 h after 602 exposure. In the present studies, at much earlier time points, we found that 602 rapidly caused autophagy-dependent degradation of HDAC proteins in sarcoma cells, as was previously observed in GI tumor cells, as well as the translocation of HDAC proteins from the nucleus to the cytosol. Our data would support the hypothesis that 602, acting as an HDAC inhibitor, would increase histone acetylation. Studies beyond the scope of the present manuscript will be required to completely understand how 602 regulates HDAC function and protein acetylation in tumor cells over hours and days.

## Data Availability Statement

The datasets presented in this article are not readily available because the data is the property of Genzada and VCU. Requests to access the datasets should be directed to Paul Dent, paul.dent@vcuhealth.org.

## Author Contributions

LB performed the studies. DH and CW provided advice and research focus. PD wrote the manuscript. All authors contributed to the article and approved the submitted version.

## Conflict of Interest

PD has received funding support from Genzada Pharmaceuticals Inc. for these studies. CW is a paid officer of the company. DH is a paid consultant and a Key Scientific advisor to the company. PD is a paid consultant and a Key Advisor for Genzada Pharmaceuticals. The remaining author declares that the research was conducted in the absence of any commercial or financial relationships that could be construed as a potential conflict of interest.

## Abbreviations

ERK: extracellular regulated kinase
PI3K: phosphatidyl inositol 3 kinase
ca: constitutively active
dn: dominant negative
ER: endoplasmic reticulum
AIF: apoptosis inducing factor
AMPK: AMP-dependent protein kinase
mTOR: mammalian target of rapamycin
JAK: Janus Kinase
STAT: Signal Transducers and Activators of Transcription
MAPK: mitogen activated protein kinase
PTEN: phosphatase and tensin homolog on chromosome ten
ROS: reactive oxygen species
CMV: empty vector plasmid or virus
si: small interfering
SCR: scrambled
IP: immunoprecipitation
VEH: vehicle
DOX: doxorubicin.
