# Corrigendum: GZ17-6.02 and Doxorubicin Interact to Kill Sarcoma Cells *via* Autophagy and Death Receptor Signaling

**DOI:** 10.3389/fonc.2021.677725

**Published:** 2021-04-16

**Authors:** Laurence Booth, Cameron West, Daniel Von Hoff, Paul Dent

**Affiliations:** ^1^ Departments of Biochemistry and Molecular Biology, Virginia Commonwealth University, Richmond, VA, United States; ^2^ Genzada Pharmaceuticals, Sterling, KS, United States; ^3^ Translational Genomics Research Institute (TGEN), Phoenix, AZ, United States

**Keywords:** autophagy, GZ17-6.02, sarcoma, doxorubicin, CD95, death receptor

In the published article, there was an error in the Conflict of Interest statement. We neglected to include that the author Paul Dent, is a paid consultant and a Key Advisor for Genzada Pharmaceuticals. The correct statement appears below.

“PD has received funding support from Genzada Pharmaceuticals Inc. for these studies. CW is a paid officer of the company. DH is a paid consultant and a Key Scientific advisor to the company. PD is a paid consultant and a Key Advisor for Genzada Pharmaceuticals. The remaining authors declare that the research was conducted in the absence of any commercial or financial relationships that could be construed as a potential conflict of interest.”

The authors apologize for this error and state that this does not change the scientific conclusions of the article in any way. The original article has been updated.

