# Supplementary material for: GZ17-6.02 and Doxorubicin Interact to Kill Sarcoma Cells via Autophagy and Death Receptor Signaling
Source: Front Oncol. 2020 Sep 2;10:1331. doi: 10.3389/fonc.2020.01331 (PMC7492267; doi:10.3389/fonc.2020.01331)

Supplemental Figure 1. Control data showing siRNA knock down of proteins and over-expression of proteins. HT1080 and MES cells, 24h after plating, were transfected with a scrambled control siRNA or with validated siRNA molecules to knock down the indicated proteins. Alternatively, cells were transfected with an empty vector plasmid or with a plasmid to over-express the indicated protein. Twenty-four h after transfection, cells were fixed in place and immunostaining performed using manufacturer validated antibodies. The percentage change in expression comparing scrambled control / CMV versus knock down siRNA or plasmid over-expression treated is indicated (n = 3 independent assessments from 100 cells per image +/- SD).

Supplemental Figure 2. Control data showing siRNA knock down of RAS proteins alongside positive control data showing neratinib reducing the expression of RAS proteins. HT1080 and MES cells, 24h after plating, were transfected with a scrambled control siRNA or with validated siRNA molecules to knock down the expression of K-RAS or N-RAS. Twenty-four h after transfection, cells were fixed in place and immunostaining performed using validated antibodies against K-RAS, N-RAS and ERK2. The expression of ERK2 was invariant. In HT1080 cells siRNA knocked down the expression of K-RAS by 70% and of N-RAS by 68%. In MES cells siRNA knocked down the expression of K-RAS by 76% and of N-RAS by 74%. As a positive control, untransfected cells were treated with vehicle control or neratinib (50 nM) for 3h, after which cells were fixed in place and immunostaining performed using validated antibodies against K-RAS, N-RAS and ERK2. In HT1080 cells neratinib reduced the expression of K-RAS by 68% and of N-RAS by 76%. In MES cells neratinib reduced the expression of K-RAS by 75% and of N-RAS by 73%.

Supplemental Figure 3. GZ17-6.02 and doxorubicin interact to activate an ATM-AMPK-ULK1-autophagy pathway concomitant with inactivation of mTOR and increased Beclin1 expression. MES human sarcoma cells were treated with vehicle control, GZ17-6.02 (2 μM final curcumin), doxorubicin (200 nM) or the drugs in combination for 3h and for 6h. Cells were fixed in place and immunostained to detect the total expression and the total phosphorylation of the indicated proteins. The phosphorylation of each phosphoprotein was corrected for total protein expression; non-phosphoproteins had their expression corrected using invariant ERK2 expression. (n = 3 +/-SD) * $p < 0.05$ less than vehicle control; # $p < 0.05$ greater than vehicle control.

Supplemental Figure 4. GZ17-6.02 and doxorubicin interact to activate c-MET. MES human sarcoma cells were treated with vehicle control, GZ17-6.02 (2 μM final curcumin), doxorubicin (200 nM) or the drugs in combination for 3h and for 6h. Cells were fixed in place and immunostained to detect the total expression and the total phosphorylation of the indicated proteins. The phosphorylation of each phosphoprotein was corrected for total protein expression; HDAC6 had its expression corrected using invariant ERK2 expression. (n = 3 +/-SD) * $p < 0.05$ less than vehicle control; # $p < 0.05$ greater than vehicle control.

Supplemental Figure 5. Knockdown of c-MET enhances [GZ17-6.02 + doxorubicin] lethality in sarcoma cells. HT1080 and MES human sarcoma cells were transfected with a scrambled siRNA or with a validated siRNA molecule to knock down the expression of c-MET. Twenty-four h after transfection, cells were treated with vehicle control or with [GZ17-6.02 (2 μM final curcumin) + doxorubicin (200 nM)] in combination for 12h. Cells were isolated, and viability determined by a trypan blue exclusion assay (n = 3 +/-SD). # $p < 0.05$ greater than corresponding value in vehicle control cells.

Supplemental Figure 6. Inhibitors of c-MET enhance the lethality of [GZ17-6.02 + doxorubicin] in sarcoma cells. HT1080 and MES cells were treated with vehicle control or with [GZ17-6.02 (2 μM final curcumin) + doxorubicin (200 nM)] in combination for 12h. In parallel cells were treated with vehicle control or with pazopanib (100 nM); crizotinib (100 nM); foretinib (100 nM); BMS-777607 (100 nM) or tivantinib (100 nM) for 12h. Cells were isolated, and viability determined by a trypan blue exclusion assay (n = 3 +/-SD). # $p < 0.05$ greater than corresponding value in vehicle treated cells.

Supplemental Figure 7. An anti-HGF neutralizing antibody prevents the drug-induced activation of c-MET; representative images. HT1080 and MES cells were treated with vehicle control or with [GZ17-6.02 (2 μM final curcumin) + doxorubicin (200 nM)] in combination for 3h and 6h in the presence of either an IgG or an anti-HGF IgG (5 μg/ml). At each timepoint cells were fixed in place and the total expression of ERK2 and c-MET determined as well as the tyrosine phosphorylation of c-MET.

Supplemental Figure 8. An anti-HGF neutralizing antibody prevents the drug-induced activation of c-MET; GRAPH. HT1080 and MES cells were treated with vehicle control or with [GZ17-6.02 (2 μM final curcumin) + doxorubicin (200 nM)] in combination for 3h and 6h in the presence of either an IgG or an anti-HGF IgG (5 μg/ml). At each timepoint cells were fixed in place and the total expression of ERK2 and c-MET determined as well as the tyrosine phosphorylation of c-MET. (n = 3 +/-SD) # $p < 0.05$ greater than vehicle control.

Supplemental Figure 9. Drug-induced alterations in protein phosphorylation require the expression of ATM. HT1080 and MES human sarcoma cells were transfected with a scrambled siRNA control (siSCR) or with a validated siRNA to knock down expression of ATM. Twenty four h afterwards, cells were treated with vehicle control or with [GZ17-6.02 (2 μM final curcumin) + doxorubicin (200 nM)] in combination for 3h. Cells were fixed in place and immunostained to detect the total expression and the total phosphorylation of the indicated proteins. The phosphorylation of each phosphoprotein was corrected for total protein expression. (n = 3 +/-SD) * $p < 0.05$ less than vehicle control; # $p < 0.05$ greater than vehicle control.

Supplemental Figure 10. Expression of activated mTOR suppresses tumor cell killing and abolishes autophagic flux. A. MES human sarcoma cells were transfected with an empty vector plasmid (CMV) or with a plasmid to express a mutant active mTOR protein. Twenty-four h afterwards, cells treated with vehicle control, GZ17-6.02 (2 μM final curcumin), doxorubicin (200 nM) or the drugs in combination for 12h. Cells were isolated, and viability determined by a trypan blue exclusion assay (n = 3 +/-SD). * $p < 0.05$ less than vehicle control. **B.** MES cells were transfected with an empty vector plasmid (CMV) or with a plasmid to express a mutant active mTOR protein and in parallel all transfected with a plasmid to express LC3-GFP-RFP. Twenty-four h afterwards, cells treated with vehicle control, GZ17-6.02 (2 μM final curcumin), doxorubicin (200 nM) or the drugs in combination for 4h or 8h. At each time point the mean number of intense GFP+ and RFP+ vesicles was determined counting > 40 cells per condition. (n = 3 +/-SD) # $p < 0.05$ greater than corresponding values after 4h; ¶ $p < 0.05$ less than corresponding values after 4h; § $p < 0.05$ less than corresponding values in CMV transfected cells.

Supplemental Figure 11. In the absence of autophagy, GZ17-6.02 -induced killing is mediated via CD95-FADD. A. MES human sarcoma cells were transfected with a scrambled siRNA (siSCR) or with an siRNA to knock down the expression of Beclin1, and in parallel, siBeclin1 transfected cells were transfected to knock down the expression of the indicated proteins. Twenty-four h afterwards, cells treated with vehicle control or with [GZ17-6.02 (2 μM final curcumin) + doxorubicin (200 nM)] in combination for 12h. Cells were isolated, and viability determined by a trypan blue exclusion assay (n = 3 +/-SD). * $p < 0.05$ less than vehicle control; ** $p < 0.05$ less than corresponding value in siBeclin1 alone. **B.** MES cells were transfected with a scrambled siRNA (siSCR) or with an siRNA to knock down the expression of Beclin1, and in parallel, cells were transfected with an empty vector plasmid or with plasmids to express BCL-XL, c-FLIP-s or dominant negative caspase 9. Twenty-four h afterwards, cells treated with vehicle control or with [GZ17-6.02 (2 μM final curcumin) + doxorubicin (200 nM)] in combination for 12h. Cells were isolated, and viability determined by a trypan blue exclusion assay (n = 3 +/-SD). * $p < 0.05$ less than vehicle control; ** $p < 0.05$ less than corresponding value in siBeclin1 alone.

Supplemental
Figure 1

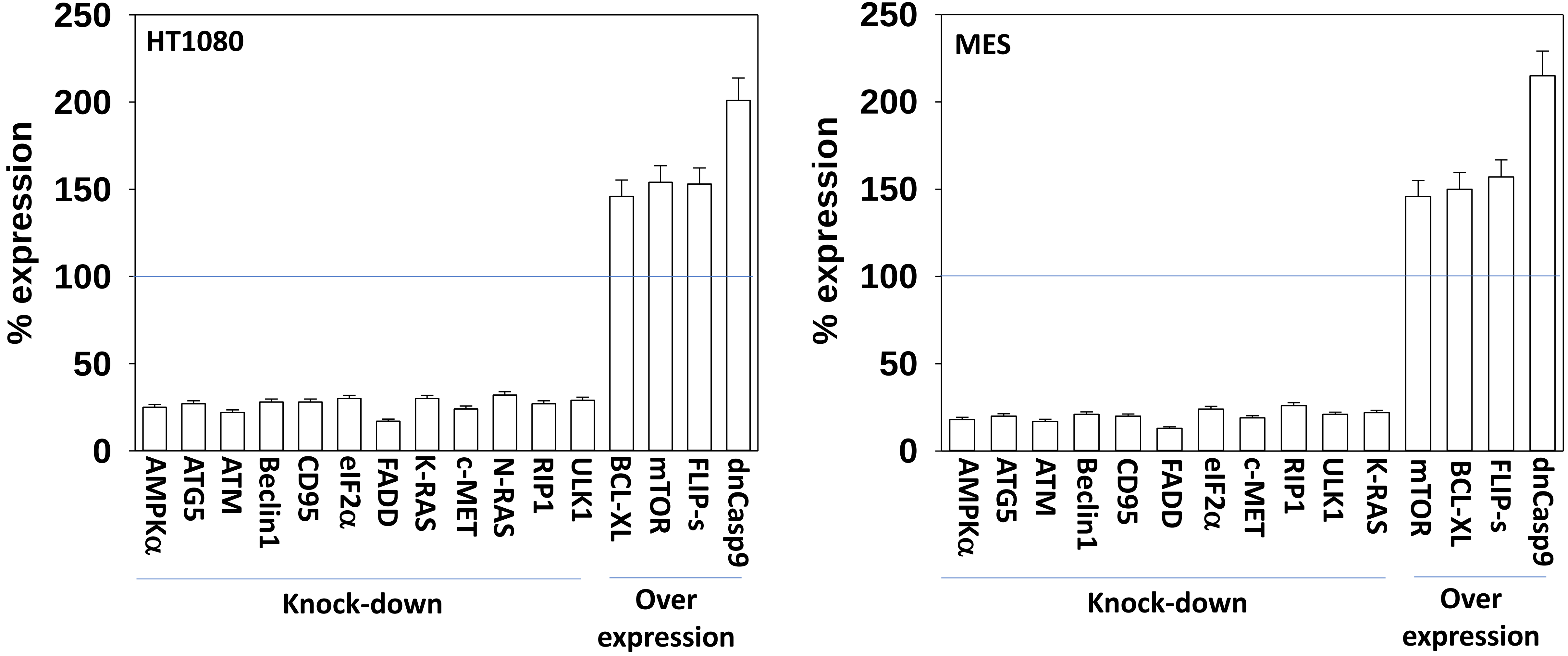

Supplemental Figure 2

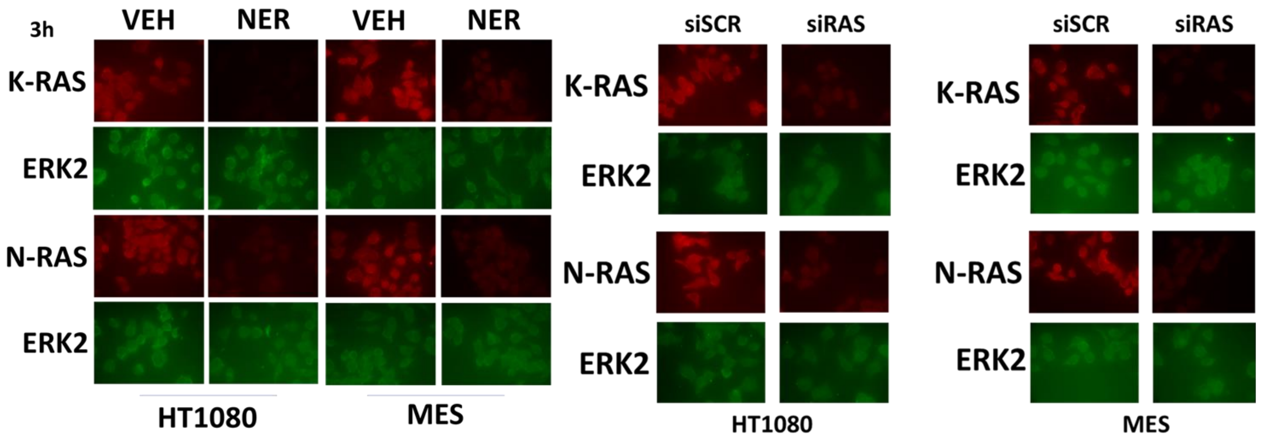

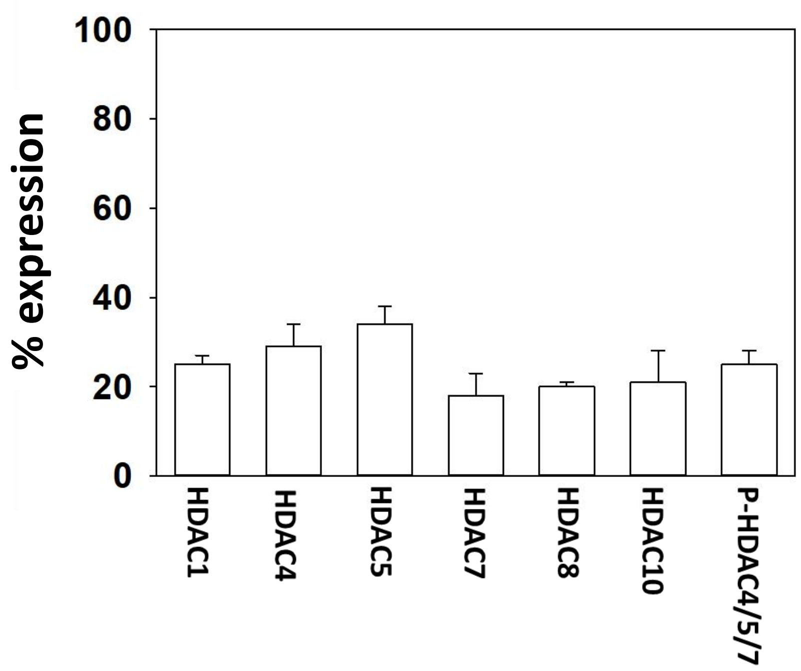

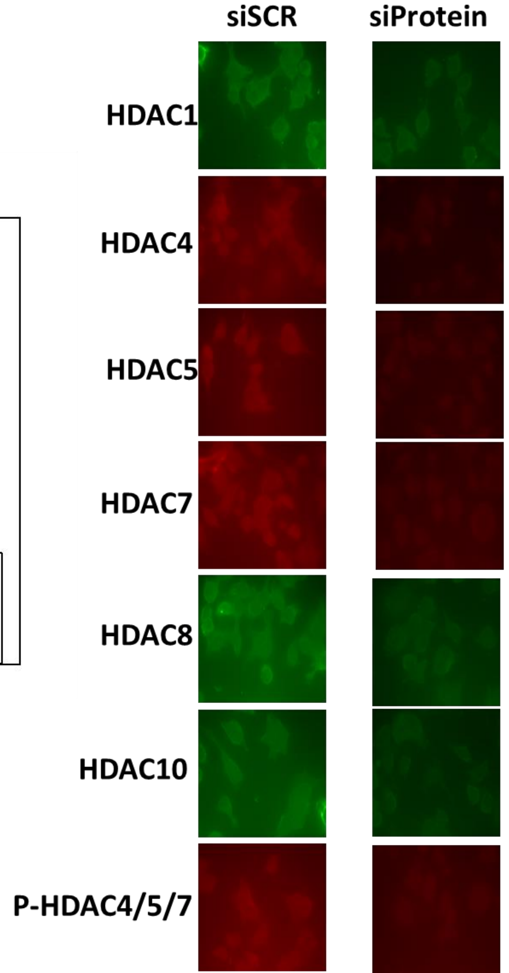

Supplemental Figure 3

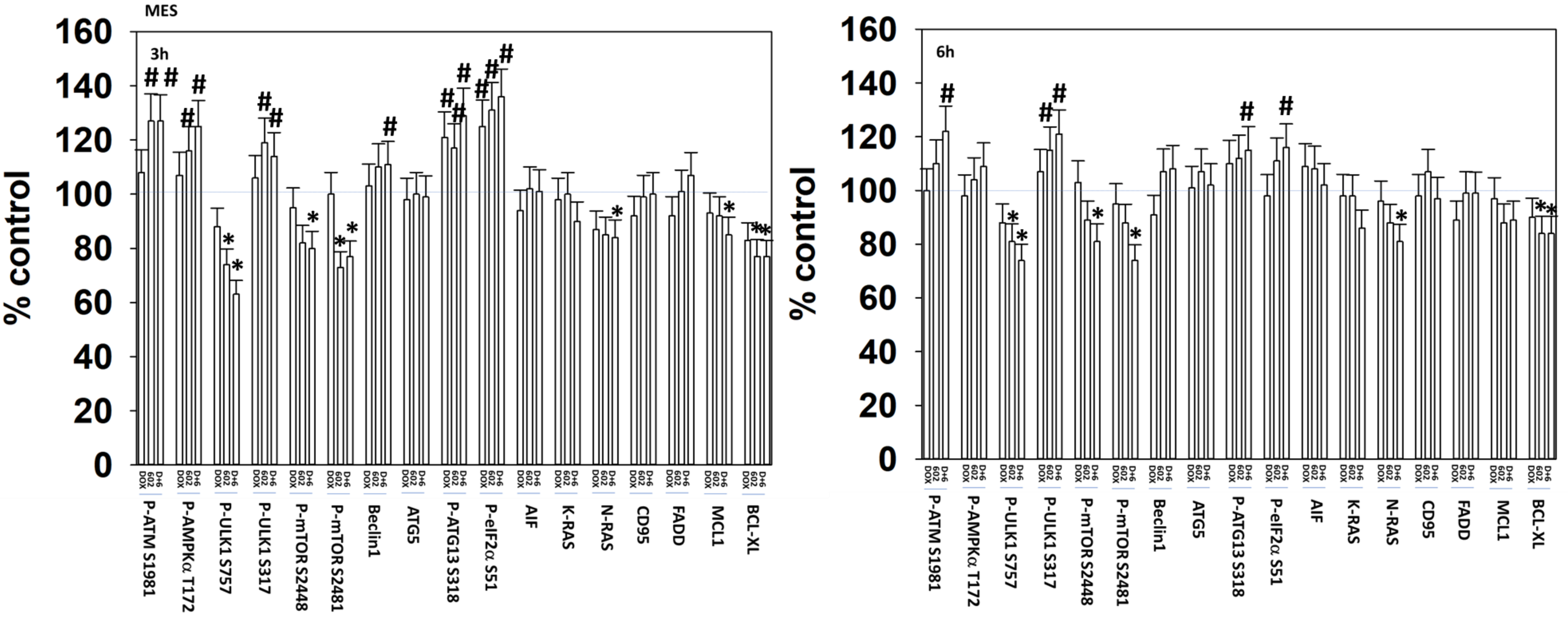

Supplemental Figure 4

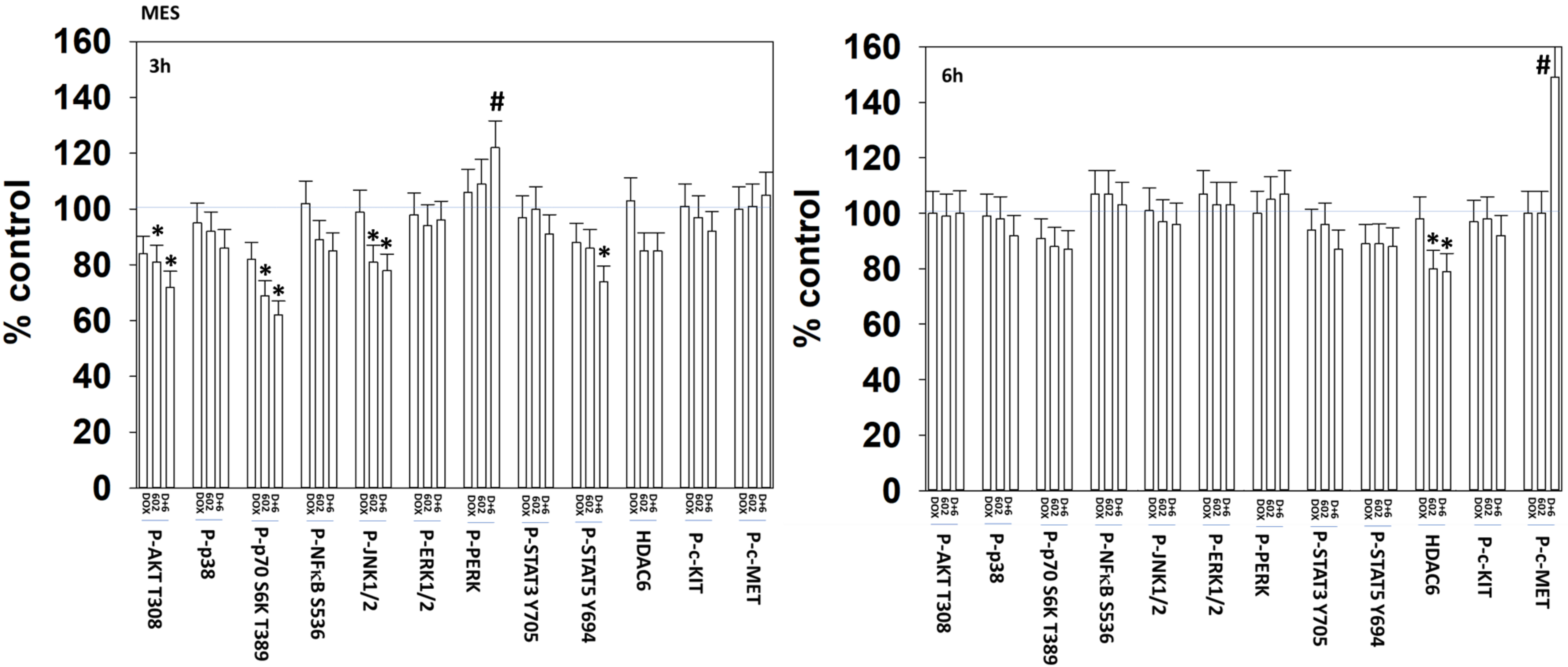

Supplemental Figure 5

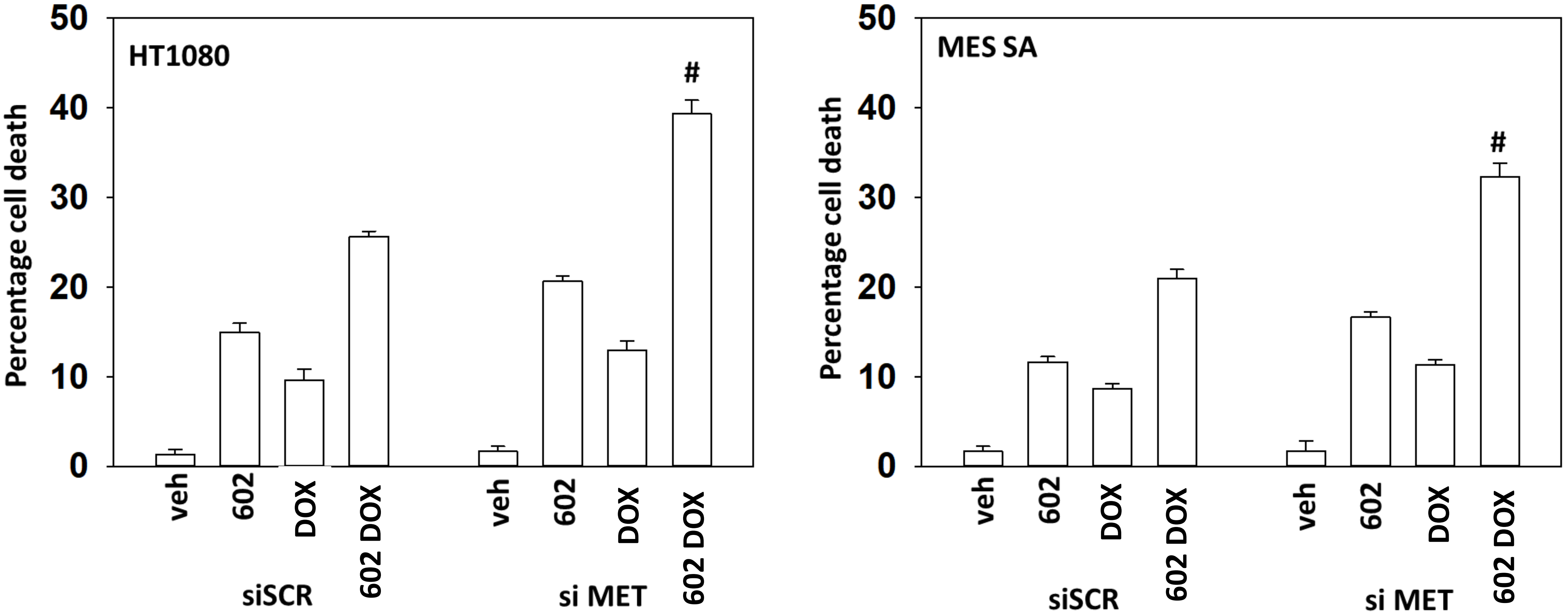

Supplemental Figure 6

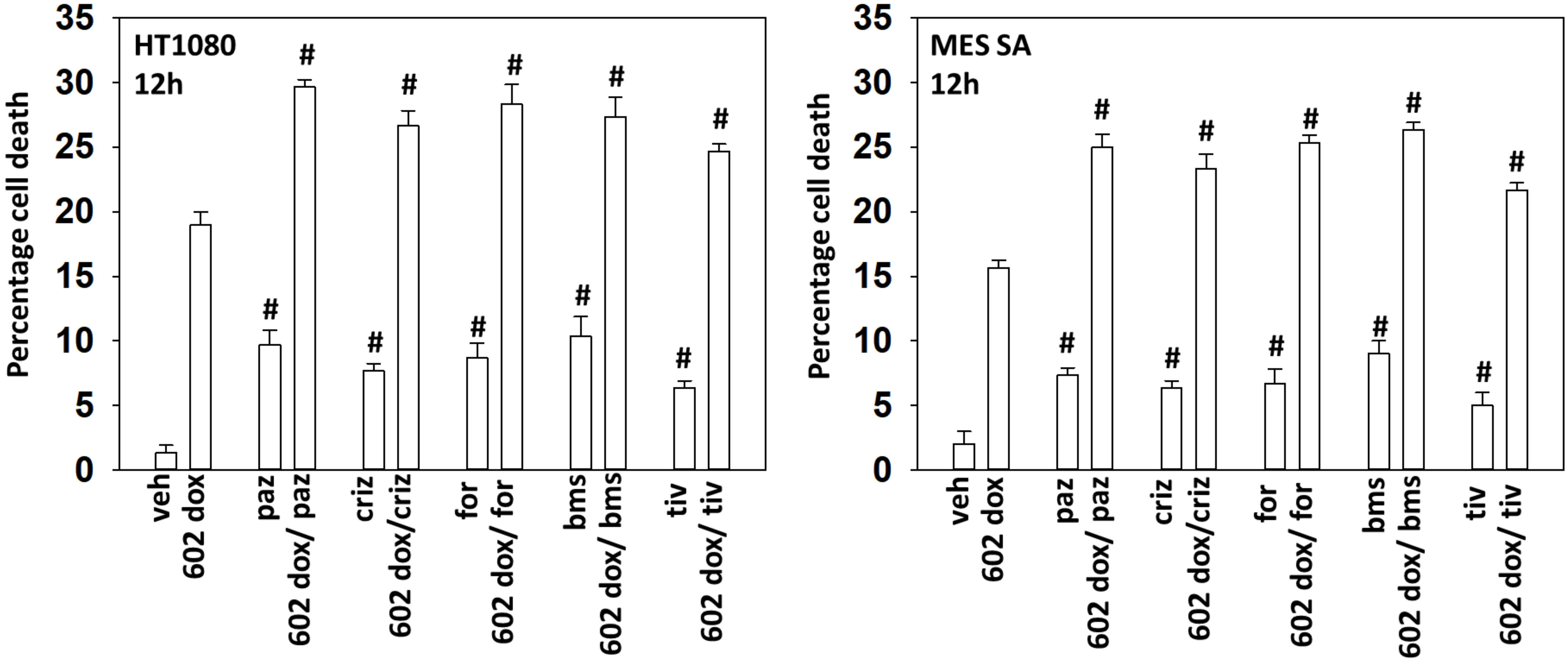

Supplemental Figure 7

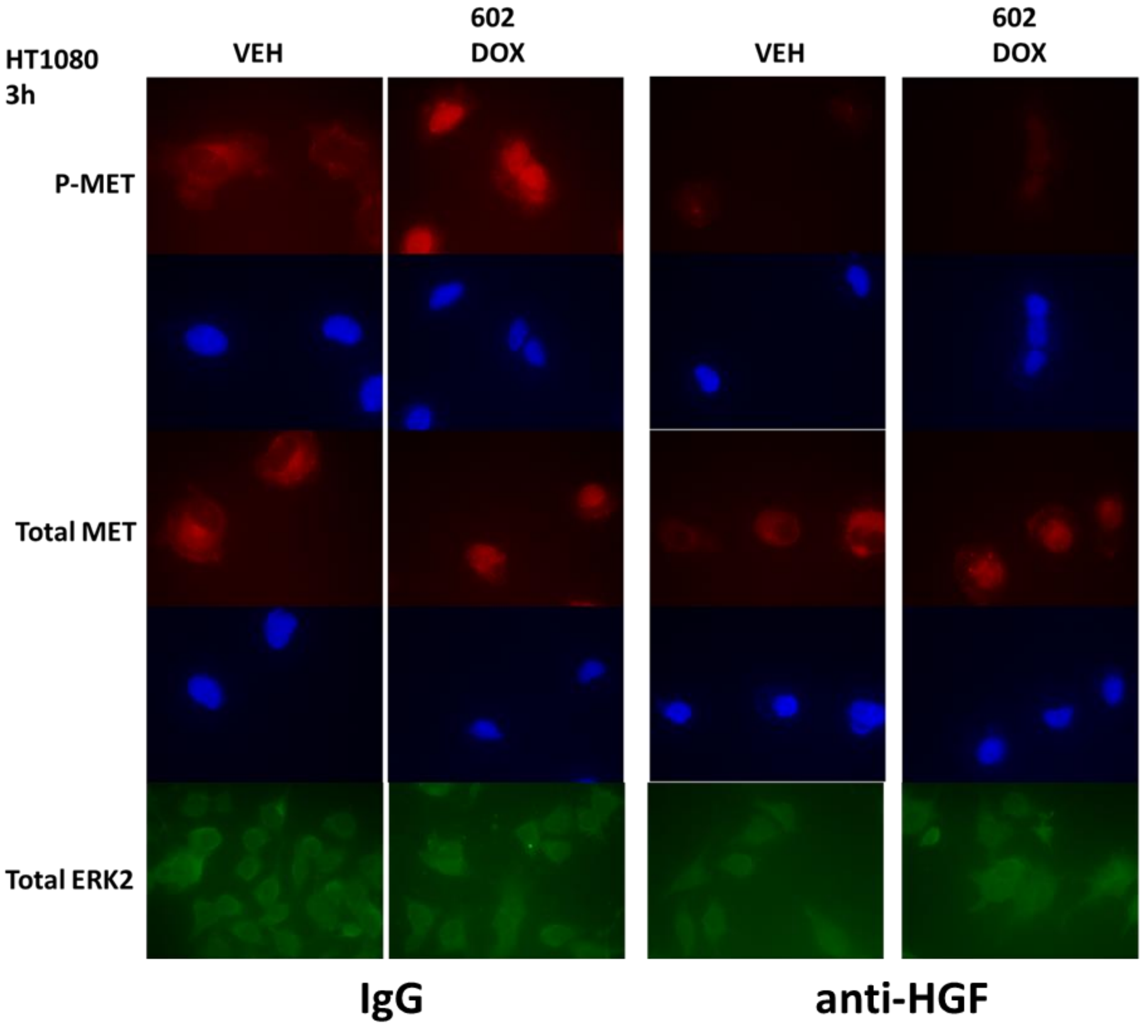

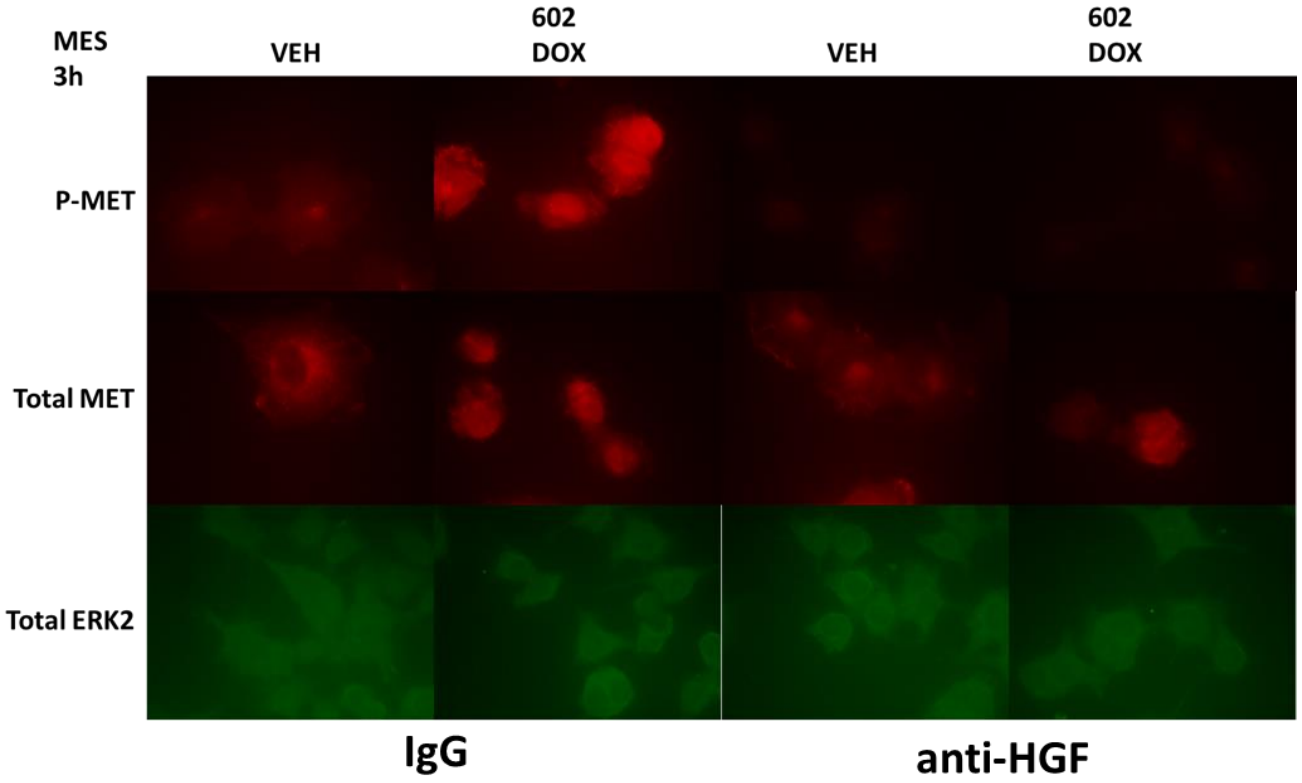

Supplemental Figure 8

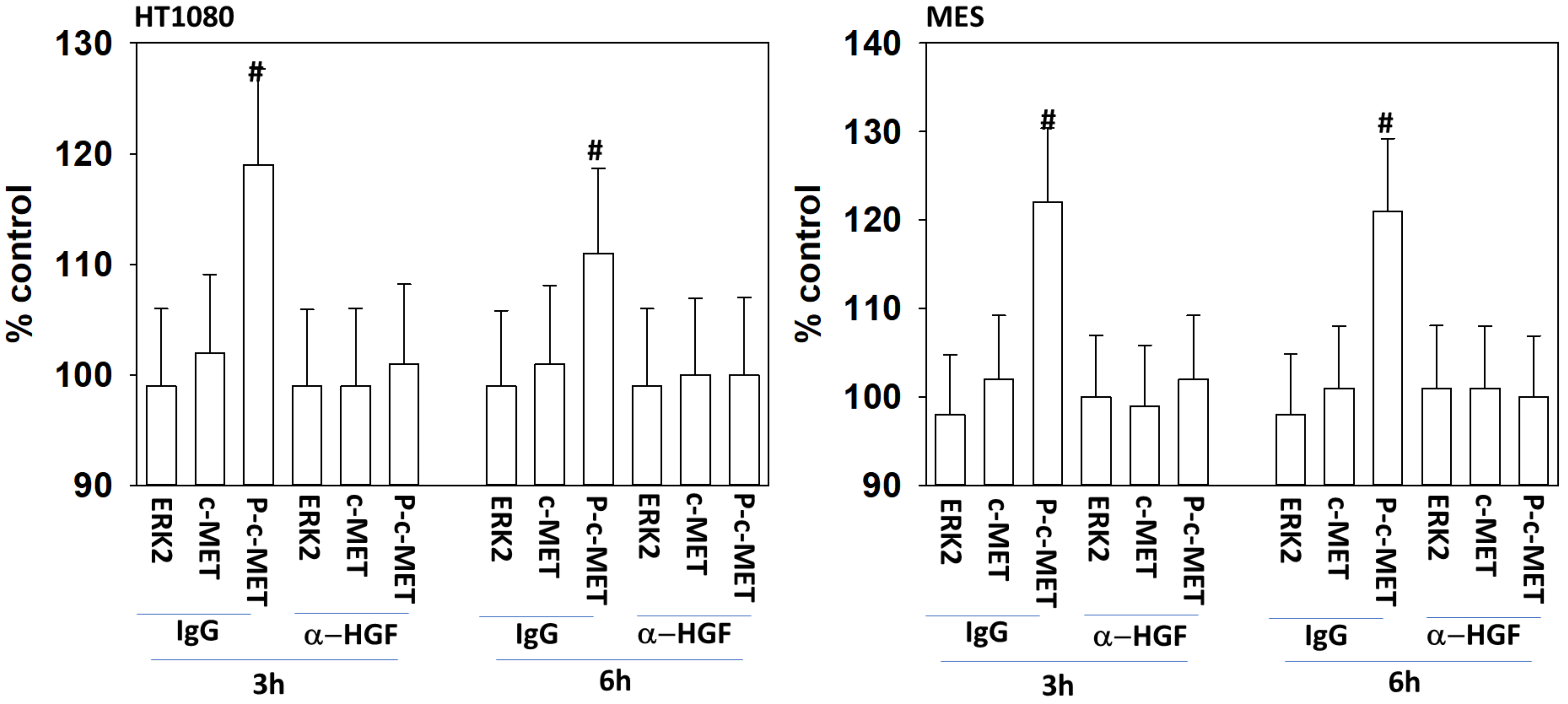

Supplemental Figure 9

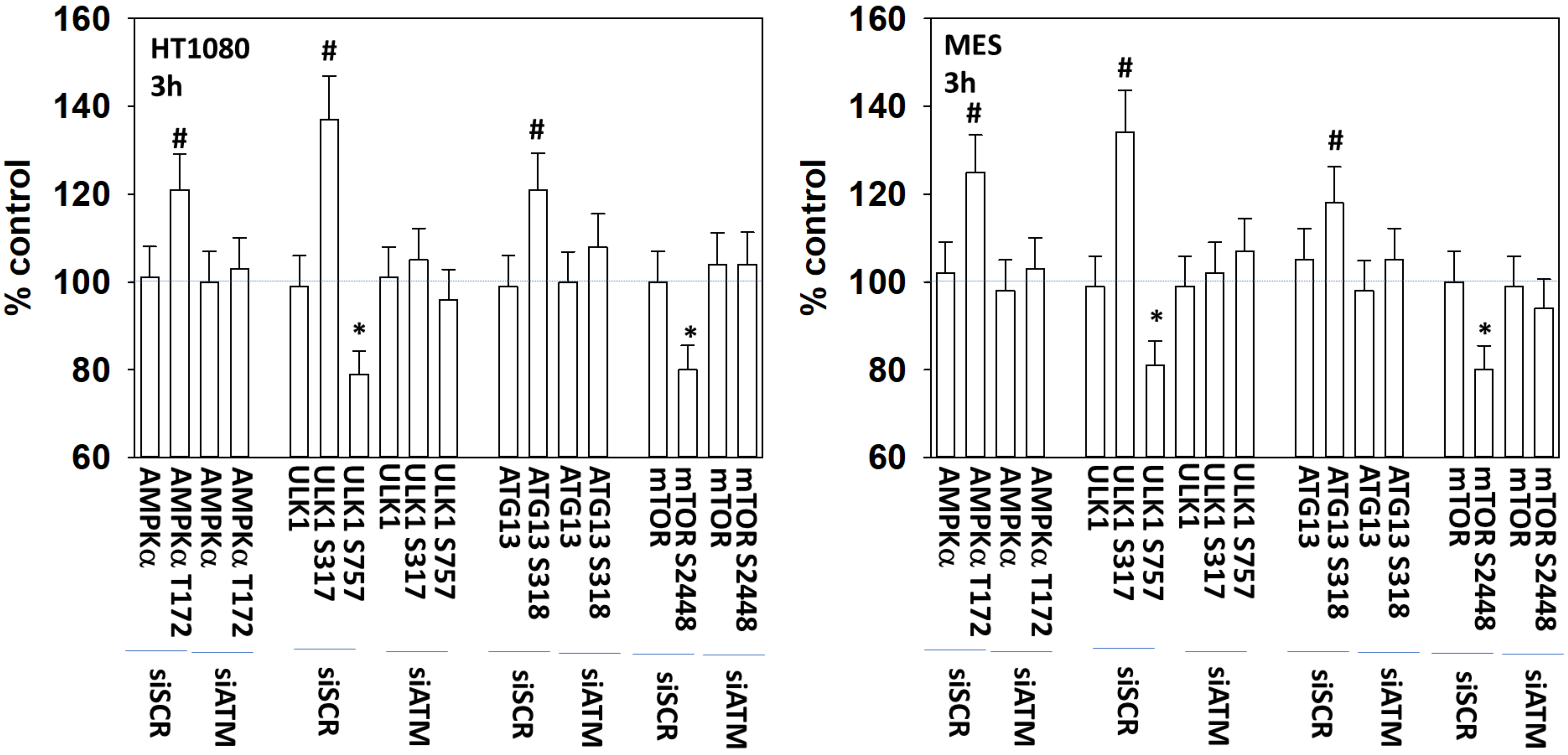

Supplemental Figure 10

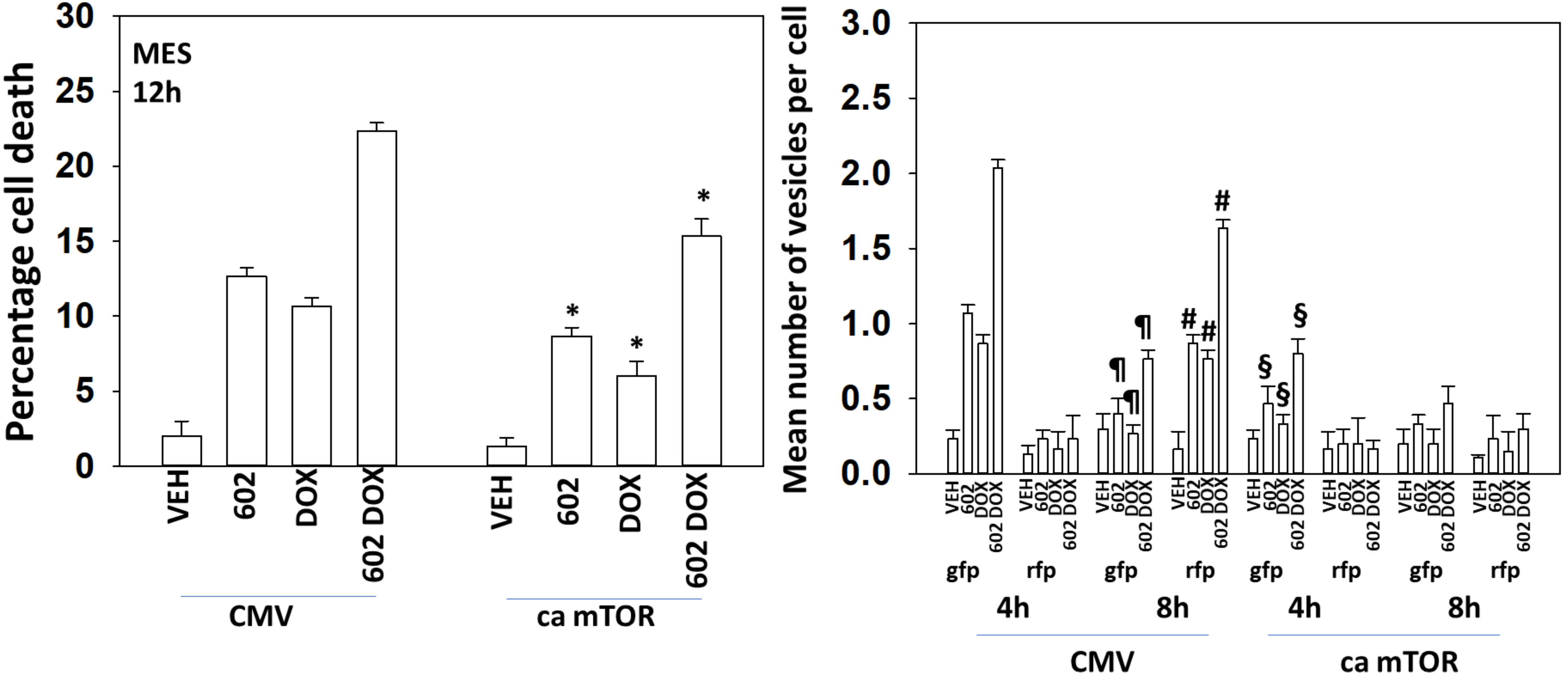

Supplemental Figure 11

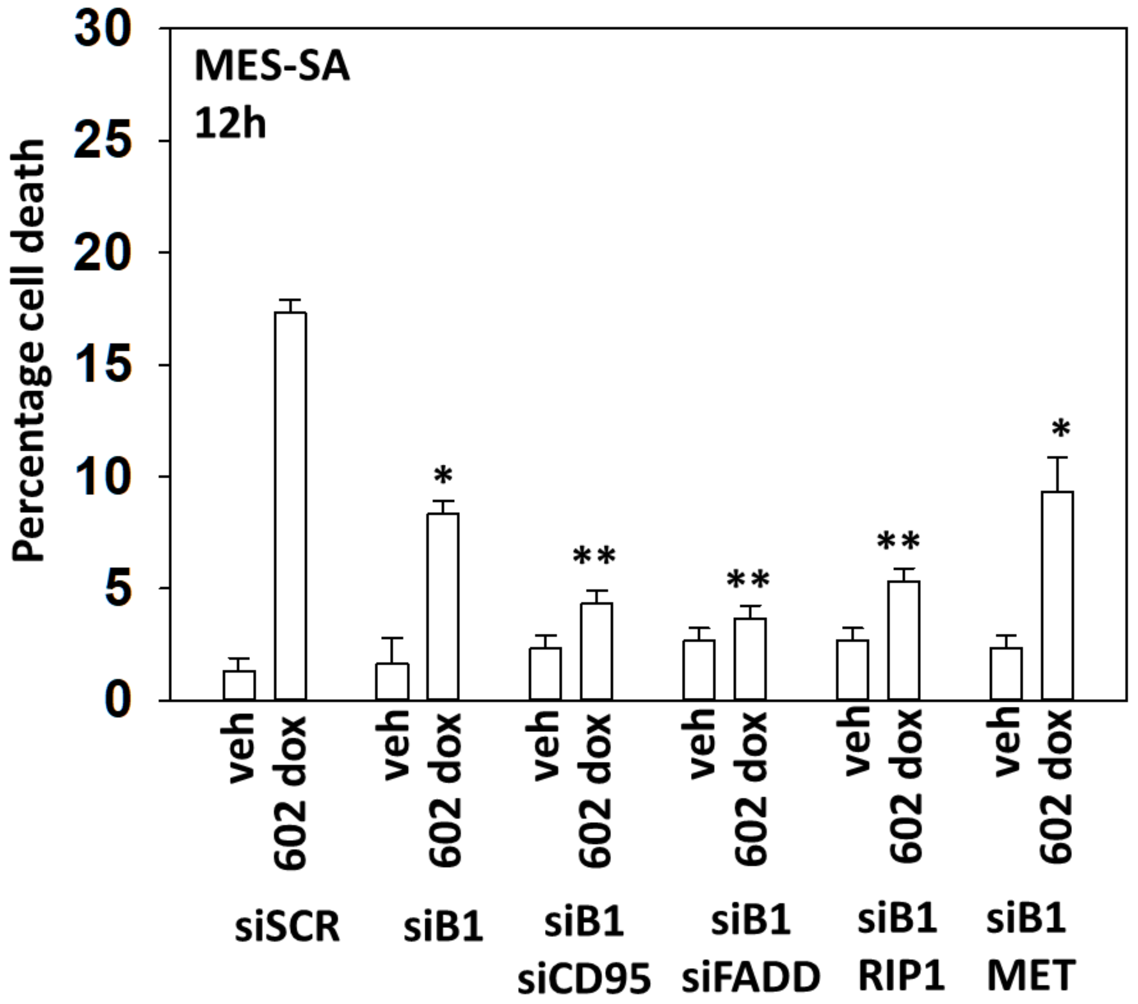

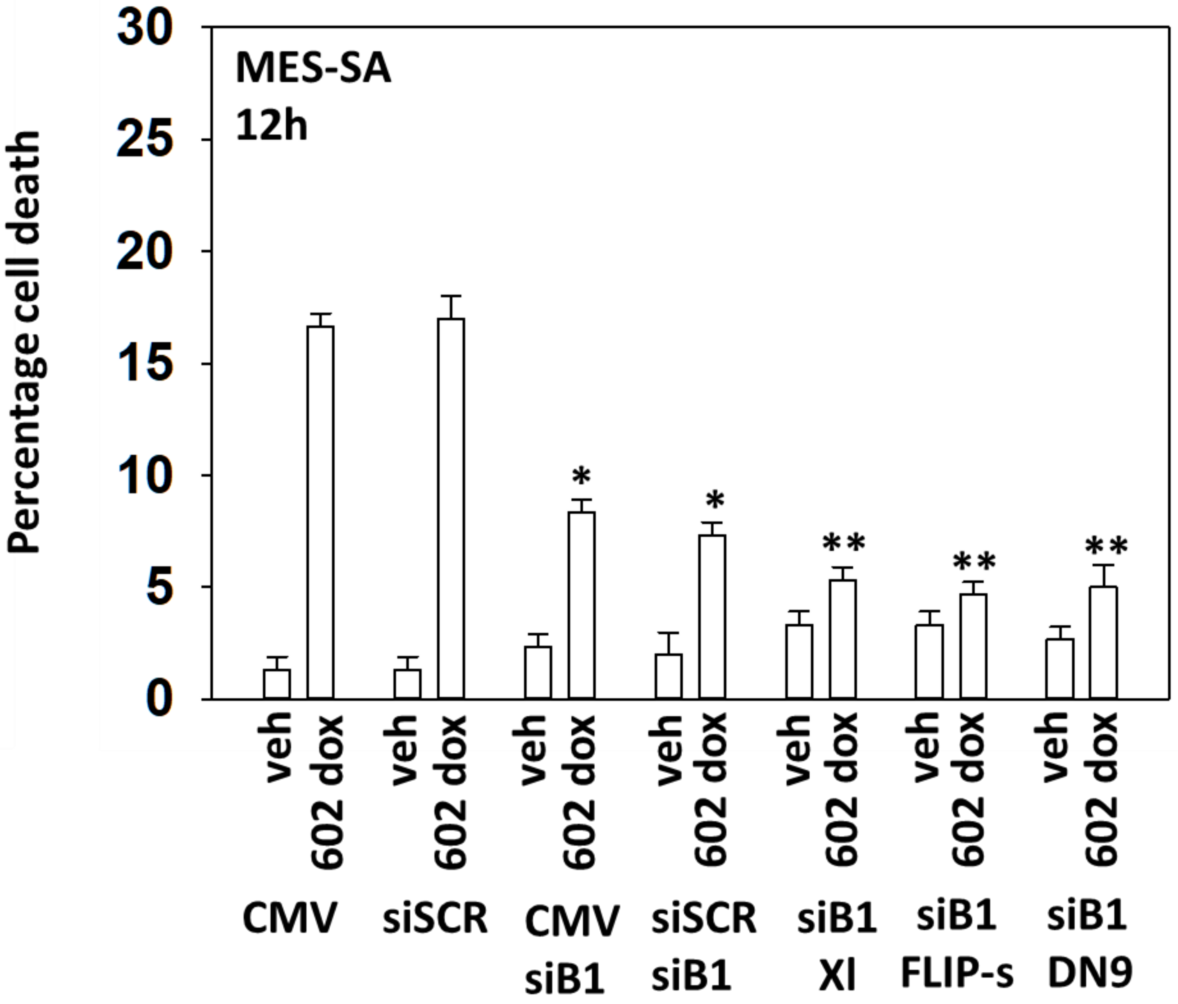

Supplement: Supplementary file 1 [file Data_Sheet_1.PDF]
